# Oral Microbiome Characteristics in Patients With Autoimmune Hepatitis

**DOI:** 10.3389/fcimb.2021.656674

**Published:** 2021-05-19

**Authors:** Benchen Rao, Jiamin Lou, Haifeng Lu, Hongxia Liang, Juan Li, Heqi Zhou, Yajuan Fan, Hua Zhang, Ying Sun, Yawen Zou, Zhongwen Wu, Yan Jiang, Zhigang Ren, Zujiang Yu

**Affiliations:** ^1^ Department of Infectious Diseases, The First Affiliated Hospital of Zhengzhou University, Zhengzhou, China; ^2^ Gene Hospital of Henan Province, Precision Medicine Center, The First Affiliated Hospital of Zhengzhou University, Zhengzhou, China; ^3^ Department of Infectious Diseases, Yiwu Central Hospital, Yiwu, China; ^4^ State Key Laboratory for Diagnosis and Treatment of Infectious Disease, The First Affiliated Hospital, School of Medicine, Zhejiang University, Hangzhou, China; ^5^ Department of Nephrology, The First Affiliated Hospital of Zhengzhou University, Zhengzhou, China; ^6^ Department of Neurology, The First Affiliated Hospital of Zhengzhou University, Zhengzhou, China

**Keywords:** autoimmune hepatitis (AIH), healthy controls (HCs), oral microbiome, microbial markers, diagnostic value

## Abstract

Autoimmune hepatitis (AIH) is a common cause of liver cirrhosis. To identify the characteristics of the oral microbiome in patients with AIH, we collected 204 saliva samples including 68 AIH patients and 136 healthy controls and performed microbial MiSeq sequencing after screening. All samples were randomly divided into discovery cohorts (46 AIH and 92 HCs) and validation cohorts (22 AIH and 44 HCs). Moreover, we collected samples of 12 AIH patients from Hangzhou for cross-regional validation. We described the oral microbiome characteristics of AIH patients and established a diagnostic model. In the AIH group, the oral microbiome diversity was significantly increased. The microbial communities remarkably differed between the two groups. Seven genera, mainly *Fusobacterium, Actinomyces* and *Capnocytophaga*, were dominant in the HC group, while 51 genera, *Streptococcus, Veillonella* and *Leptotrichia*, were enriched in the AIH group. Notably, we found 23 gene functions, including Membrane Transport, Carbohydrate Metabolism, and Glycerolipid metabolism that were dominant in AIH and 31 gene functions that prevailed in HCs. We further investigated the correlation between the oral microbiome and clinical parameters. The optimal 5 microbial markers were figured out through a random forest model, and the distinguishing potential achieved 99.88% between 46 AIH and 92 HCs in the discovery cohort and 100% in the validation cohort. Importantly, the distinguishing potential reached 95.55% in the cross-regional validation cohort. In conclusion, this study is the first to characterize the oral microbiome in AIH patients and to report the successful establishment of a diagnostic model and the cross-regional validation of microbial markers for AIH. Importantly, oral microbiota-targeted biomarkers may be able to serve as powerful and noninvasive diagnostic tools for AIH.

## Introduction

Autoimmune hepatitis (AIH) is an inflammation of the liver parenchyma mediated by an autoimmune response to liver cells. A majority of patients with AIH gradually develop cirrhosis, relapsing disease, hepatic failure or death. Although the correlations between AIH and genetic or environmental factors have been widely accepted to some extent, the exact etiology of AIH has not yet been fully illustrated (Wang et al., 2016). The etiology and clinical characteristics of AIH are different from other autoimmune liver diseases such as primary sclerosing cholangitis (PSC) and primary biliary cirrhosis (PBC), AIH has become an increasingly important public health concern (Anand et al., 2019).

Although the diagnostic criteria of AIH have been reported, there are certain confounders, such as AIH induced by drugs, autoantibody-negative AIH, features of AIH shared with PBC or PSC in overlap syndrome (OS), and posttransplant AIH (Wei et al., 2020).

Recently, rapid developments in the study of microbiota have emphasized the crucial role that the microbiota plays in the pathogenesis, diagnosis or therapy of numerous diseases. A growing number of experts are showing increasing interest in the microbial field. Oral dysbiosis not only contributes to oral diseases such as periodontitis, dental caries and oral mucosal diseases but also relates to systemic diseases, including gastrointestinal system diseases such as inflammatory bowel disease (IBD) (Said et al., 2014), liver cirrhosis (LC) (Bajaj et al., 2015), pancreatic cancer (Ren et al., 2017), nervous system diseases such as Alzheimer’s disease (AD) (Aguayo et al., 2018), endocrine system diseases such as diabetes (Xiao et al., 2017), adverse pregnancy outcomes (Corwin et al., 2017), obesity and polycystic ovary syndrome (Akcali et al., 2014), immune system diseases such as rheumatoid arthritis (RA) (Zhang et al., 2015) and cardiovascular system diseases such as atherosclerosis(AS) (Koren et al., 2011).

A large number of published studies have shown that oral and gut microbes play important roles in the development of many liver diseases. Dysbiosis has been found in the oral and gut microbiome of patients with Chronic hepatitis B (Lu et al., 2011; Ling et al., 2015), liver cirrhosis (Bajaj et al., 2015), PSC (Lapidot et al., 2021) and hepatocellular carcinoma (HCC) (Lu et al., 2016; Ren et al., 2019). Our previous study has described the gut microbiomes of AIH patients (Lou et al., 2020). Another previous study involved 17 AIH salivary samples that described the oral microbiome of AIH patients (Abe et al., 2018). However, the oral microbiomes of AIH patients have not, though, hitherto been subject to scientific investigation, and there is a lack of adequate systematic randomized controlled trials in this field (Zhang et al., 2015; Gao et al., 2018). Therefore, our study of oral microorganisms in the field of AIH is well-founded and necessary. Moreover, regional differences are important influencing factors for gut microbiota differences (Gopalakrishnan et al., 2018; Caussy et al., 2019). This study described the characteristics of the oral microbiome of AIH patients from central China and clarified the effectiveness of the oral microbiome as a diagnostic tool for AIH.

## Materials and Methods

### Participant Information

The study was designed and conducted according to the PRoBE (prospective specimen collection and retrospective blinded evaluation), the Helsinki Declaration, and the Rules of Good Clinical Practice. Ethical clearance was obtained from the Ethical Review Board of the First Affiliated Hospital of Zhengzhou University (No. 2017-XY-002). Written consent of each participant has been obtained (Deschasaux et al., 2018).

All saliva samples were collected from the First Affiliated Hospital of Zhengzhou University. And all participants were in the outpatient with newly diagnosed AIH. The diagnostic criteria for AIH were as follows: the score of 1999 International AIH Group ≥ 10, (2) the simplified score of 2008 International AIH Group ≥ 6 and characteristic AIH histology. All the participants in this study were newly diagnosed patients. Participants were excluded if they merged the following reasons: ​(1) overlap syndrome (OS), (2) primary biliary cholangitis (PBC), (3) antibiotic, proton pump inhibitors, steroids or UDCA consumption within the past 8 weeks, and (4) other liver diseases (alcoholic liver disease, viral hepatitis and nonalcoholic steatohepatitis) (Sebode et al., 2018; Wei et al., 2020).

In this study, a total of 185 saliva samples including 85 AIH patients and 100 HCs were prospectively collected, and after the strict screening, 69 patients with AIH and 99 age-, sex-, and BMI-matched HCs from the physical examination center were enrolled. Eventually, 16S rRNA MiSeq sequencing was conducted by 168 saliva samples from AIH patients and HCs.

Participants’ demographics and clinicopathological data were collected from hospital electronic medical records and questionnaires **(** **Table 1** **)**.

**Table 1:** Clinical characteristics of the cohorts.

Characteristics	Discovery cohort			Validation cohort			
	AIH (n=46)	HC (n=92)	P value*	AIH (n=22)	HC (n=44)	P value*	P value^†^
Demographics							
Age, years, median(min–max) IOR	52(39-69) 9	52.5(25-72) 5	0.586	49.5(25-72) 11	50(28-77) 7	0.897	0.108
Gender, Female,n(%)	41(89.13%)	80(86.96%)	0.714	18(81.81%)	34(77.27%)	0.667	0.415
BMI,kg/m2, median(min–max) IQR	21.435 (18.37-23.91) 2.213	21.975 (18,37-24,57) 2.497	0.067	21.32 (18.471-23.81) 2.455	21.23 (18.52-23.44) 2.698	0.391	0.932
Hepatic function, median (min-max) IQR							
ALT, U/L	145(47-553) 97	15(7-38) 6	0.000	135.5(56-415) 103	19(7-99) 13	0.000	0.338
AST, U/L	96.5(40-407) 66	19(11-33) 5	0.000	101(47-301) 53	21(14-36) 7	0.000	0.546
AKP, U/L	69(40-263) 22	70.5(32-157) 31	0.575	61(50-351) 26	63.5(32-157) 35	0.295	0.332
GGT, U/L	89(54-321) 40.5	18(6-64) 11.25	0.000	95(54-120) 38.5	14(7-80) 10.05	0.000	0.365
TB, umol/L	21(6-89) 14.5	12(4-30) 5.25	0.000	33.5(9-98) 17	12(6-29) 6.5	0.000	0.088
ALB, g/L	38.5(29-54) 11	48.25(39.6-55.2) 4.5	0.000	42.5(29-48) 9	48.05(40.6-53.3) 6.5	0.000	0.108
Immunoglobulin, median (min–max) IQR							
IgG, g/L	21.39(9.82-44) 5.39			21.9(18.3-25.3) 3.62			0.605
IgM, g/L	1.55(0.51-5.3) 1.32			1.6(0.8-3.2) 0.93			0.777
IgA, g/L	2.15(0.6-8.63) 0.69			3.2(1.2-4.2) 0.85			0.159
Autoantibody, +/-, +%							
ANA	42/4,91.3			20/2,90.91			0.975
ASMA	10/36,21.74			4/18,18.18			0.732
SLA/LP	9/37,19.57			3/19,13.64			0.541

*Comparisons between AIH and controls.

^†^Comparisons between AIH in exploration cohort and those in the validation cohort.

Use SPSS v. 20.0 (IBM Corp., Armonk, NY, USA) to analyze the data. Calculate the statistical significance of the differences between groups. Compare categorical variables by Fisher’s exact test. Compare continuous variables by the Wilcoxon rank-sum test. Correlation analysis was conducted by Spearman’s rank test.

BMI, body mass index; AIH, autoimmune hepatitis; ALT, alanine aminotransferase; AST, aspartate aminotransferase; AKP, alkaline phosphatase; GGT, gamma-glutamyltransferase; TB, total bilirubin; ALB, albumin; IgG, Immunoglobulin G; IgM, Immunoglobulin M; IgA, Immunoglobulin A; ANA, antinuclear antibody; ASMA, anti-smooth muscle antibody; SLA/LP, soluble liver antigen/liver pancreas antigen; IQR, interquartile range.

### Saliva Sample Collection and DNA Extraction

Saliva samples should be collected as soon as possible at the time of enrollment to avoid medical intervention factors which may cause changes in the saliva microbiome. Collect and pretreat saliva samples as previously reported (Bajaj et al., 2015). In order to maintain good oral hygiene, participants were asked to brush their teeth every morning and night. Before donating saliva, each participant was instructed to fast for two hours. 5 ml saliva was needed and spat into the saliva collection tube. We stored the samples were at −80°C as soon as possible, we excluded samples which were at room temperature for >2 hours. Anyone participating in the study did not consume probiotics, antibiotics, cigarettes or drugs within eight weeks before enrollment.

DNA extraction was performed with genomic DNA extraction kit as previously reported by us (Ren et al., 2019).

### PCR Amplification and MiSeq Sequencing

We used the forward primer 5’-ACTCCTACGGGAGGCAGCA-3’ and the reverse primer 5’-GGACTACHVGGGTWTCTAAT-3’ for PCR amplification. PCR amplification was carried out in a 20-μL reaction system which consisted 10 ng of template DNA, 0.4 μL of TransStart Fastpfu DNA polymerase (TransGen Biotech, Beijing, China), 0.4 μL of reverse primer (5 μM), 0.4 μL of forward primer (5 μM), 2 μL of 2.5 mM dNTPs and 4 μL of 5× Fastpfu buffer. The ABI GeneAmp 9700 (Thermo Fisher Scientific, Waltham, MA, USA) was used to conduct PCR as we previously reported (Ren et al., 2019).

Mix the purified PCR products and construct DNA libraries in accordance with the manufacturer’s instructions. Shanghai Mobio Biomedical Technology Co., Ltd., Shanghai, China performed the sequencing by an Illumina MiSeq Platform (Ren et al., 2013).

### Sequence Data Processing

Assign screened readings to varieties of samples according to specific barcodes before removing barcodes and primers. Use FLASH v. 1.2.10 to overlap the default parameters with the paired-end sequenced reads of each library (Magoc and Salzberg, 2011). Conduct the quality control on overlapping readings generated by LASH. Use UCHIME v. 4.2.40 to detect and remove the chimeric sequences (Edgar et al., 2011). The operational taxonomic units (OTUs) were matched by the Broad Institute 16S database(microbiome util-r20110519 version; http://drive5.com/uchime/gold.fa). In addition, the nucleotide sequences of all samples were submitted to the European Nucleotide Archive (ENA) database (PRJNA557511).

### OTU Clustering and Taxonomic Annotation

We randomly select an equal number of readings from all samples, then binned the OTUs with the UPARSE pipeline (Edgar, 2013).

We counted total OTUs at different levels including phylum, class, order, family and genus. The OTU serial numbers of all samples are displayed in the statistical table (Kakiyama et al., 2013).

### Bacterial Diversity and Taxonomic Analysis

Use the ‘vegan’ R package to calculate the Simpson and Shannon indices for bacterial community diversity. Use the Ace and Chao estimators to evaluate the richness of the bacterial community. In order to compare microbial community richness, we constructed rarefaction curves. Reveal the similarity and overlap of OTUs through Venn diagrams and identify common and unique OTUs in multiple samples. Heatmaps were drawn using Heatmap Builder to describe the main species. Through species composition analysis, we generated the microbial community bar plots (Ren et al., 2014).

NMDS and Principal coordinates analysis (PCoA) analysis were conducted using the ‘vegan’ R package to identify the microbial space between samples (Ren et al., 2017). Use the ‘phyloseq’ package to identify the unweighted and weighted UniFrac distances. The Spearman correlation analysis was conducted to figure out the correlation between the oral microbiome and clinic indicators. Describe the evolutionary relationship of bacteria through phylogenetic trees. Perform bacterial taxonomic analyses at different levels (in the order of phylum, class, order, family, and genus). Then, we conducted Wilcoxon rank-sum tests to compare the microbiome difference between the two groups. Use the linear discriminant analysis effect size (LEfSe) method to conduct A linear discriminant analysis (LDA) to figure out key microbiomes with significant differences (http://huttenhower.sph.harvard.edu/lefse/e/). The cutoff value was set as an LDA score of log10 = 2 (Lu et al., 2017). Moreover, the Wilcoxon rank-sum test and the nonparametric Kruskal-Wallis rank-sum test were used to identify key biomarkers (community members).

### Gene Function Prediction

The gene functions of the oral microbiome and 16S rRNA gene sequences in KEGG, COG, and Rfam were predicted by PICRUSt. (Lu et al., 2019).

In order to predict the metabolic functions of bacterias, the 16S rRNA gene sequencing data was compared with the database with known metabolic functions by PICRUSt. Taking into account the relative differences in the copy number of 16S rRNA genes between species, we have corrected the abundance data of the original species to improve the accuracy and reliability of the prediction (Wang et al., 2018).

### OTU Biomarker Identification and POD Index Construction

In order to figure out significantly different OTUs in the two groups, we used the random forest 4.6–12 package to construct a random forest model by the R 3.4.1 software. Then, a five-fold cross-validation was conducted to evaluate the generalization error (Fouhy et al., 2019). Then, plot the cross-validation error curve. The point with the lowest cross-validation error was set as the cutoff point. In addition, the optimal OTUs set was the set with the fewest OTUs (Ren et al., 2018).

POD index was defined as the ratio between the number of randomly generated decision trees that predicted sample as ‘AIH’ and that of HCs. We plotted the ROC curve and calculated the area under curve to evaluate the diagnostic efficiency of the established model by the pROC package (Tilg et al., 2018).

### Statistical Analysis

Use SPSS v. 20.0 (IBM Corp., Armonk, NY, USA) to analyze the data. Calculate the statistical significance of the differences between groups. Compare categorical variables by Fisher’s exact test. Compare continuous variables by the Wilcoxon rank-sum test. Correlation analysis was conducted by Spearman’s rank test.

## Results

### Characteristics of the Participants

**Figure 1:**
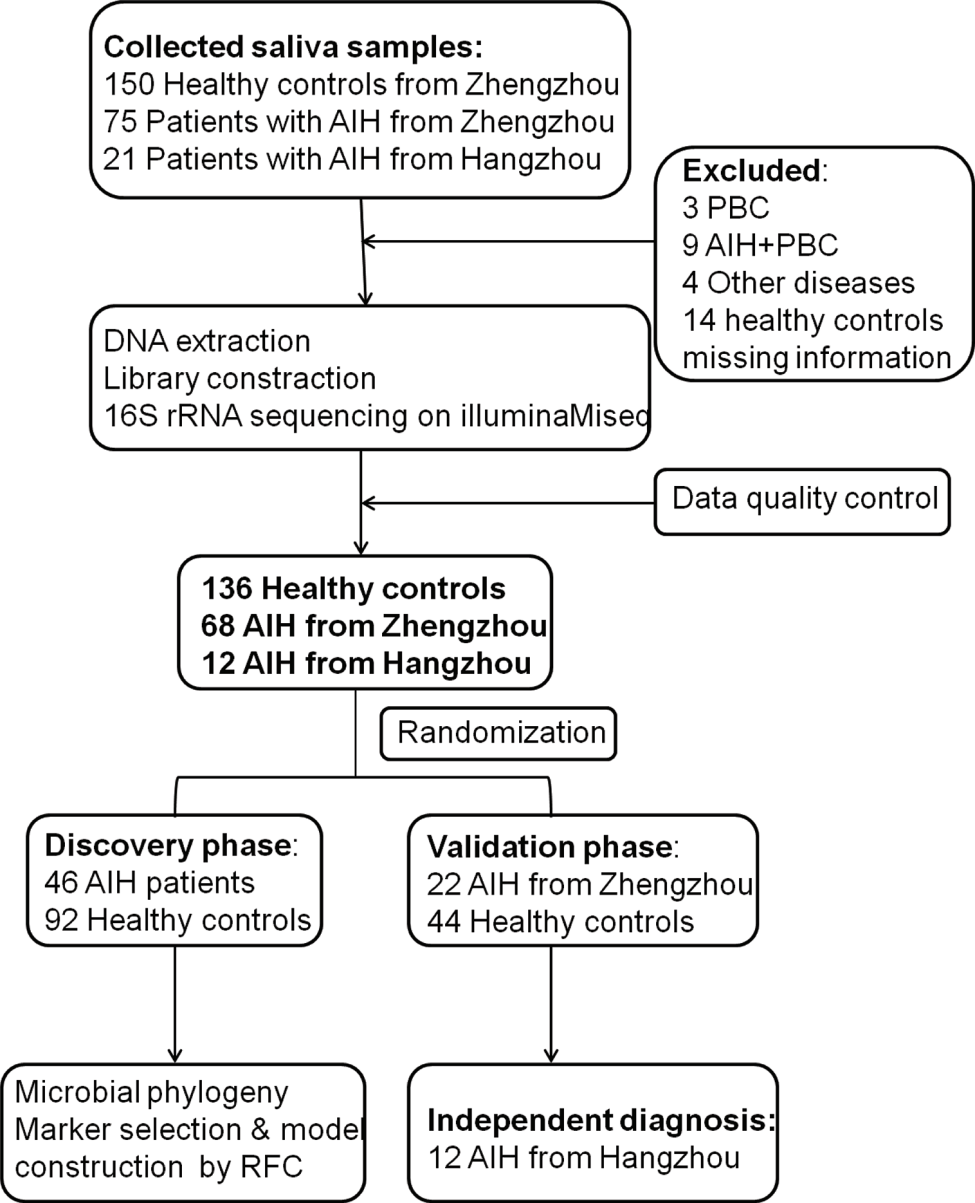
Study design and flow diagram. A total of 225 saliva samples from Central China were prospectively collected. After a strict pathological diagnosis and exclusion process, 68 AIH patients and 136 HCs were included and randomly divided into the discovery phase and validation phase. In the discovery phase, saliva microbiome characteristics were described between 46 AIH and 92 HCs. And microbial markers were figured out, then a diagnostic model between AIH and HCs was constructed by a random forest model. In the validation phase, 44 HCs and 22 AIH patients were used to validate the diagnostic efficacy of the AIH classifier. During the independent diagnostic phase, 12 AIH patients from Hangzhou and 44 HCs were included in the study for cross-regional validation. AIH, autoimmune hepatitis; HCs, healthy controls.

A total of 204 saliva samples from Central China were prospectively collected. 68 newly diagnosed patients with AIH and 136 BMI-, gender-, and age-matched HCs were included and randomly divided into the discovery phase and validation phase after a strict selection and exclusion process **(** **Figure 1** **)**. In the discovery phase, we characterized the saliva microbiome between 46 AIH patients and 92 HCs, figured out microbiome markers and constructed an AIH classifier tool for AIH by a random forest model. In the validation phase, 44 HCs and 22 AIH patients were used to validate the diagnostic efficacy of the AIH classifier. Moreover, we collected saliva samples from 12 AIH patients from Hangzhou with 44 HCs for cross-regional validation during the independent diagnostic phase. The detailed demographic and clinical characteristics of the participants, including gender, age, BMI, hepatic function and immunoglobulin, of both the AIH group and the control group in the discovery phase along with the validation phase are presented in **Table 1**.

In both the discovery phase and the validation phase, most AIH patients were middle-aged and elderly women. Just over 80% of the AIH patients were female in both the discovery phase and validation phase, and the median age was 52 in the discovery phase and 49.5 in the validation phase. Clinical characteristics, including age, gender and BMI, were matched between the AIH and HC groups (P > 0.05) to minimize differences from other reasons. The liver function indices such as total bilirubin (TB), γ-glutamyltransferase (GGT), aspartate aminotransferase (AST) and alanine aminotransferase (ALT) significantly increased in AIH patients than these in the HCs (P < 0.05). While serum albumin (ALB) significantly decreased in the AIH group (P < 0.05). The serum level of immunoglobulin, IgG, in particular, was increased over 21.0 g/L in AIH patients. More than 90% of AIH patients were characterized by positive ANA (91.3% of discovery phase and 90.91% of validation phase). Moreover, in terms of comparisons between AIH in the discovery phase and those in the validation phase, there was no significant difference (P>0.05) in age, gender, BMI, hepatic function, immunoglobulin or autoantibodies.

### The Increased Oral Microbial Diversity in AIH

First, the rarefaction curve (**Supplementary Figure S1**) and rank-abundance curve (**Supplementary Figure S2**) shows that the sequencing data of the sample are reasonable and can reflect the vast majority of microbial information in the sample. The average number of reads of AIH group was 35664.34 ± 4124.39, and the average number of reads was 43477.39 ± 11137.57 in the control group.

**Figure 2:**
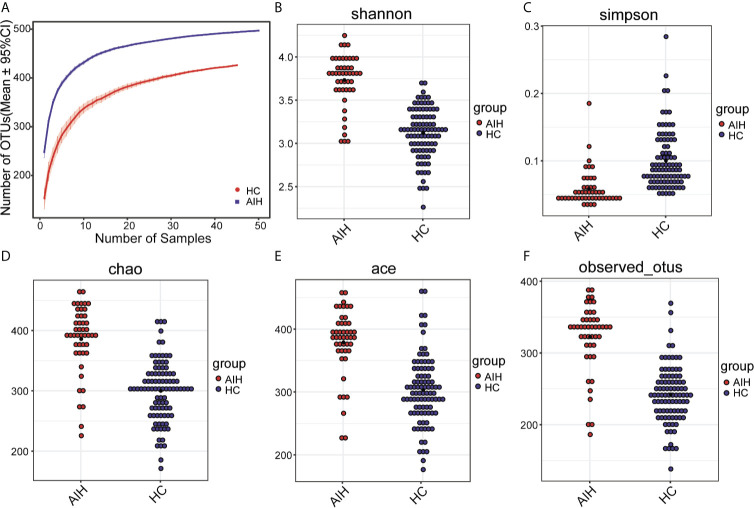
The microbial diversity was increased in AIH patients (N=46) versus HCs (N=92). **(A)** Specaccum species accumulation curves indicated the sufficient sampling size. Compared with HCs (blue), oral microbial diversity, which was calculated by **(B)** the Shannon index and **(C)** Simpson index, was significantly increased in AIH patients (red) (P<0.001, Mann-Whitney U test). Both the **(D)** Chao index and **(E)** Ace index illustrated that AIH patients (red) were characterized by higher microbial community richness than HCs (blue) (P<0.001, Mann-Whitney U test). The findings were confirmed through **(F)** the observed OTU dot plot (P<0.001, Mann-Whitney U test). The details of the indices above are listed in **Supplementary Data S1.** Plot parameters. The ‘black dot’ symbol represents the median value. AIH, autoimmune hepatitis; HCs, healthy controls.

As seen from thespecies accumulation curves **(** **Figure 2A** **)**, the curves turned into a slowly rising asymptote after the initial sharp rise, indicating that the sampling size was sufficient for the data analysis. Compared with HCs, oral microbial diversity, which was calculated by the Shannon index **(** **Figure 2B** **)** and Simpson index **(** **Figure 2C** **),** was significantly increased in AIH patients in the discovery phase (P<0.001, Mann-Whitney U test). In addition, both the Chao index **(** **Figure 2D** **)** and Ace index **(** **Figure 2E** **)** illustrated that AIH patients were characterized by higher microbial community richness than HCs (P<0.001, Mann-Whitney U test). The findings were confirmed through the observed OTU dot plot **(** **Figure 2F** **)** (P<0.001, Mann-Whitney U test). The details of the indices above are listed in **Supplementary Data S1**.

### Differences Between AIH and Healthy Individuals in the Oral Microbiome

**Figure 3:**
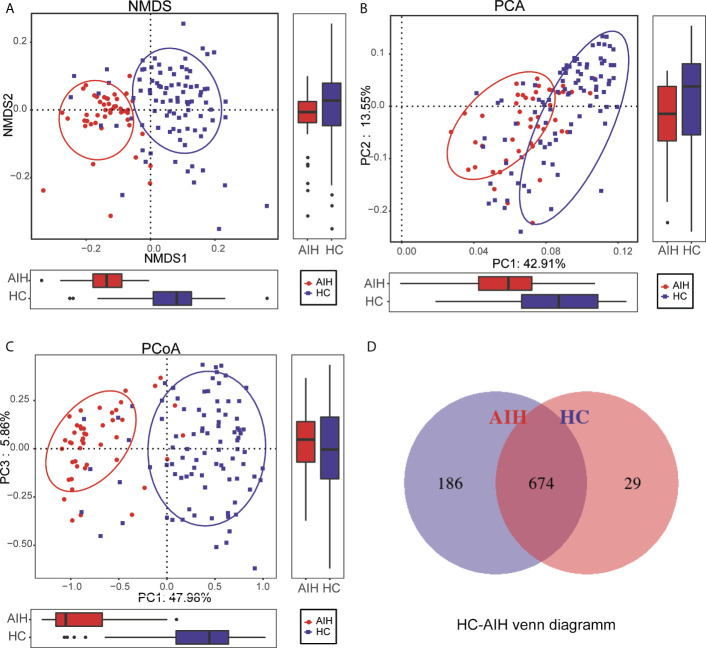
Comparisons of beta diversity between AIH patients (N=46) and HCs (N=92). The significant difference was found between AIH group and HCs group by **(A)** NMDS analysis, **(B)** PCA and **(C)** PCoA, which indicated that the composition of the overall oral microbiota of AIH and HCs was different. **(D)** 674 of the 889 OTUs were shared between AIH group and HCs group by a Venn diagram. HCs, healthy controls; AIH, autoimmune hepatitis; OTUs, operational taxonomic units; NMDS, nonmetric multidimensional scaling; PCA, principal component analysis; PCoA, principal coordinate analysis.

To display the microbiome space between samples, beta diversity was calculated through nonmetric multidimensional scaling (NMDS) analysis, principal component analysis (PCA) and principal coordinate analysis (PCoA). NMDS analysis of unweighted UniFrac **(** **Figure 3A** **),** PCA of unweighted UniFrac PC1-2 **(** **Figure 3B** **)** and PCoA of unweighted UniFrac PC1-3 **(** **Figure 3C** **)** displayed that the samples of AIH and HCs were observably separated in the direction of the NMDS1, PC1 and PC1 axes, indicating that the overall oral microbial composition was different between AIH and HCs. The single most striking observation to emerge from the data comparison was a significant difference in oral microbial communities between AIH patients and HCs. **Supplementary Data S2–S5** showed the details of the NMDS, PCA and PCoA analyses respectively.

In addition, 674 of the 889 OTUs were shared between AIH goups and HCs group by a Venn diagram **(** **Figure 3D** **)**. Notably, 186 of 889 OTUs were unique for AIH. The details of the HC-AIH Venn diagram are shown in **Supplementary Data S6**.

### Operational Taxonomic Unit (OTU) Clustering and Taxonomic Analysis

The phylogenetic tree of the OTU phyla, displayed in **Supplementary Figure S3**, described the systematic evolutionary relationships of the 12 phyla.

**Figure 4:**
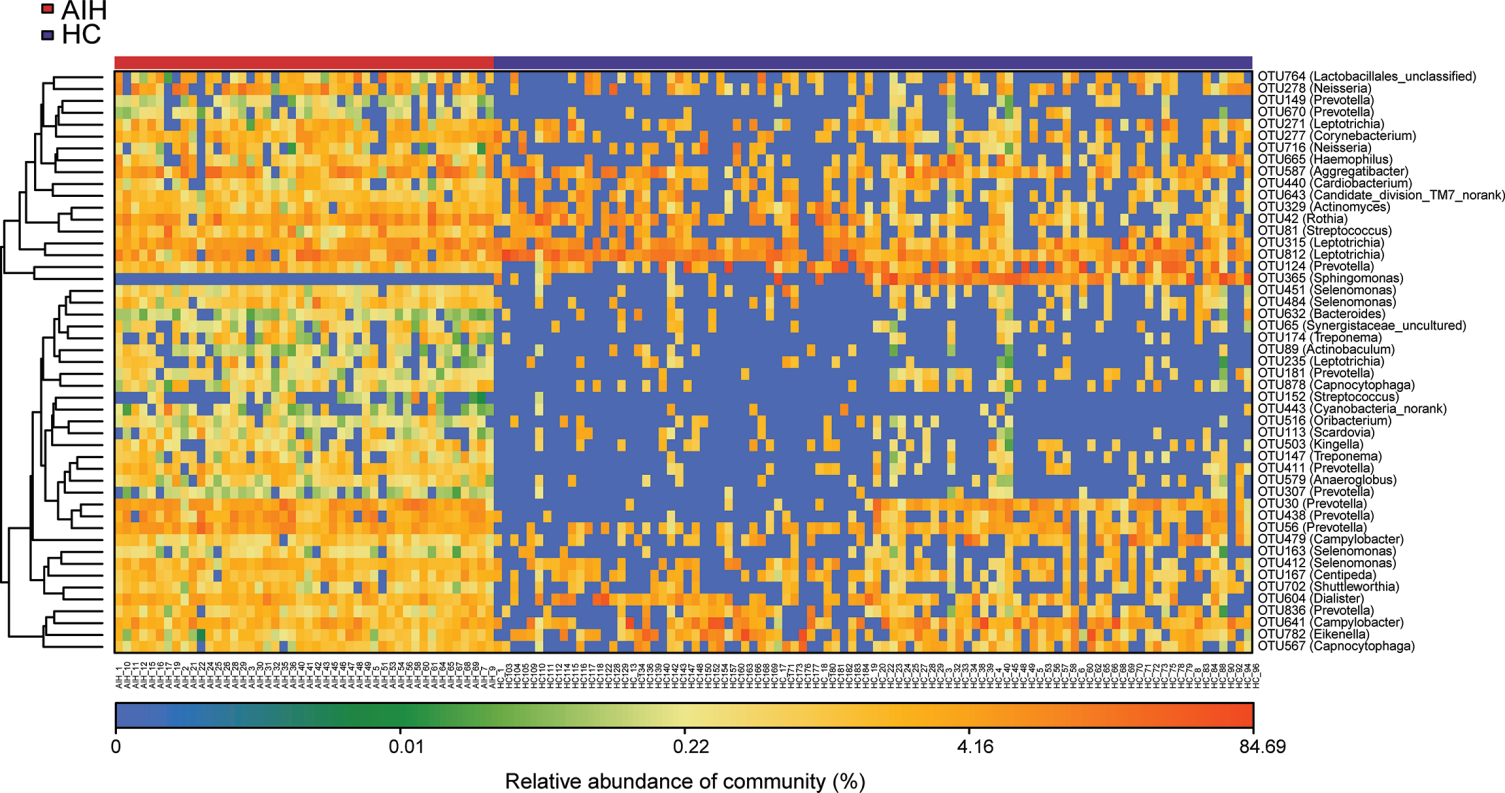
The heatmap showed the difference in the relative abundance of OTUs between AIH (N = 46) and HCs (N = 92). The relative abundance of each sample was shown. (Red means high abundance, and blue means low abundance). Each row represents one OTU. OTUs, operational taxonomic units; AIH, autoimmune hepatitis; HCs, healthy controls.

Furthermore, as shown in the heat map of the relative abundances of the discrepant OTUs between the two groups **(** **Figure 4** **)**, the closer the color is to blue, the lower the relative abundance of each OTU is. In contrast, the closer the color is to red, the higher the relative abundance of each OTU is.

A total of 22 OTUs, including OTU278 (Neisseria), OTU764 (Lactobacillales_unclassified), OTU149 (Prevotella), OTU451 (Selenomonas) and OTU315 (Leptotrichia), were enriched in AIH patients. Nevertheless, OTU365 (Sphingomonas) decreased remarkably in the oral microbiome of AIH patients compared with HCs. The above results are presented in **Supplementary Data S7**.

### Composition and Comparison of the Oral Microbiome in AIH Patients and HCs

In regard to the composition of oral microbiomes in both AIH patients and HCs, according to the annotation of OTUs, the relative abundance of each sample was calculated and plotted at each taxonomic level.

**Supplementary Figures S4** and **S5** showed the microbial relative abundance at the phylum level and genus level for each sample. Details of the microbial community at the phylum level and genus level are presented in **Supplementary Data S8** and **S9**.

**Figure 5:**
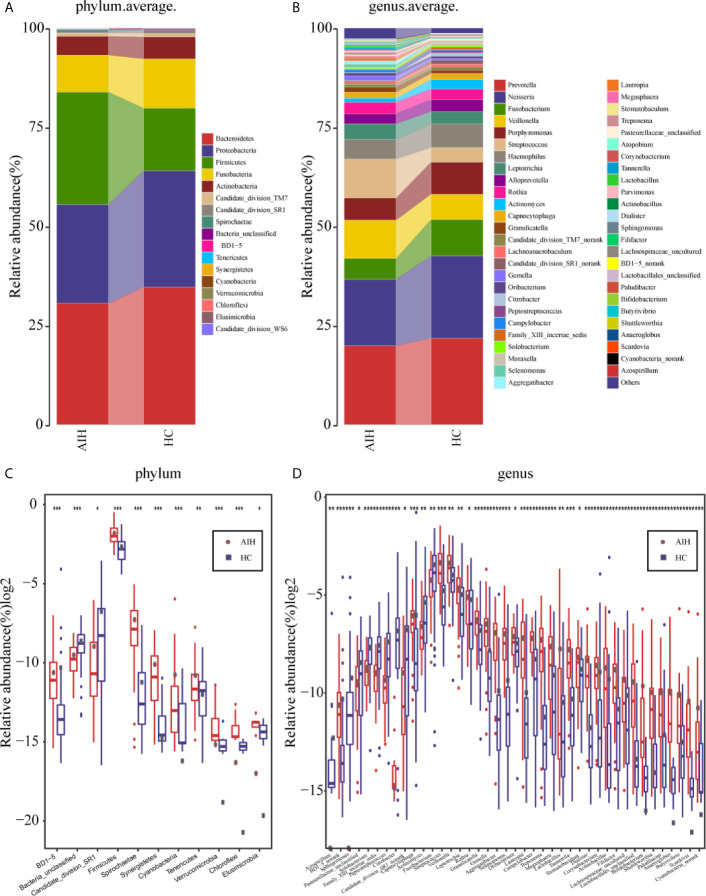
Composition and comparison of the oral microbiome in AIH patients (N=46) and HCs (N=92). **(A)**The phylum level and **(B)**genus level composition diagrams showed the composition characteristics of the two groups of oral microbiome. The differences in the relative abundance of key bacterias in the two groups were compared at **(C)** the phylum level and **(D)** the genus level. The relative abundance of each bacteria was represented by the mean ± SE. We used the Wilcoxon rank-sum test to evaluate whether the difference of relative abundance was significant (*P < 0.05; **P < 0.01 and ***P < 0.001). AIH, Autoimmune hepatitis; HCs, healthy controls.

The average proportion of *Bacteroidetes*, *Proteobacteria*, *Firmicutes*, *Fusobacteria* and *Actinobacteria* in the two groups was up to 90% at the phylum level **(** **Figure 5A** **)**. Surprisingly, significant divergences of those five main phyla were observed between the two groups. Likewise, at the genus level, 12 genera, including *Prevotella*, *Neisseria*, *Fusobacterium*, *Veillonella*, *Porphyromona*s and *Streptococcus*, accounted for an average of more than 80% in both groups **(** **Figure 5B** **)**. At the phylum level and genus level, the microbial composition of AIH patients was distinct from that of HCs.

Subsequently, a comparison of the oral microbiome in AIH (N=46) and HCs (N=92) at each taxonomic level was performed. We used the Wilcoxon rank-sum test to analyze the significant difference in microbial composition between the two groups, and corrected the p-value to the q-value by the false discovery rate (FDR). At the phylum level, on the one hand, the abundance of 9 phyla, *Firmicutes*, *Spirochaetae*, *Tenericutes*, *Synergistetes* and *Cyanobacteria*, in AIH patients was significantly higher than that in HCs (P<0.005). On the other hand, *Candidate_division_SR1* and *Bacteria_unclassified* were remarkably reduced in AIH (P<0.05) **(** **Figure 5C** **** and **Supplementary Data S10** **)**.

At the genus level, the abundances of 29 genera, such as *Treptococcus, Veillonella, Leptotrichia, Rothia, Granulicatella* and *Gemella*, were significantly higher in AIH patients than in HCs (P<0.05), while the abundances of 12 genera, including *Fusobacterium, Capnocytophaga, Actinomyces* and *Solobacterium*, decreased evidently in AIH patients (P<0.05) **(** **Figure 5D** **** and**Supplementary Data S11** **)**.

The comparison at the phylum and genus levels revealed that there was a significant difference (P <0.05) between the two groups in terms of some phyla or genera, especially *Streptococcus, Veillonella, Leptotrichia, Rothia* and *Firmicutes*. Similar results were also concluded at the class level **(** **Supplementary Figure S6A** **)**, order level **(** **Supplementary Figure S6B** **)** and family level **(** **Supplementary Figure S6C** **)**, and the P values were far below the 0.05 level **(** **Supplementary Figures S7** **–** **S9** and **S12** **–** **S14** **)**.

### Phylogenetic Characteristics of Oral Microbial Communities in AIH

According to Lefse analysis and LDA Score based on OTUs characterizing microbiota between AIH and HCs, 51 genera were certified to be deferential species for AIH. Meanwhile, 7 genera were considered to be dominant in HCs, and the difference between the two groups was of high significance.

**Figure 6:**
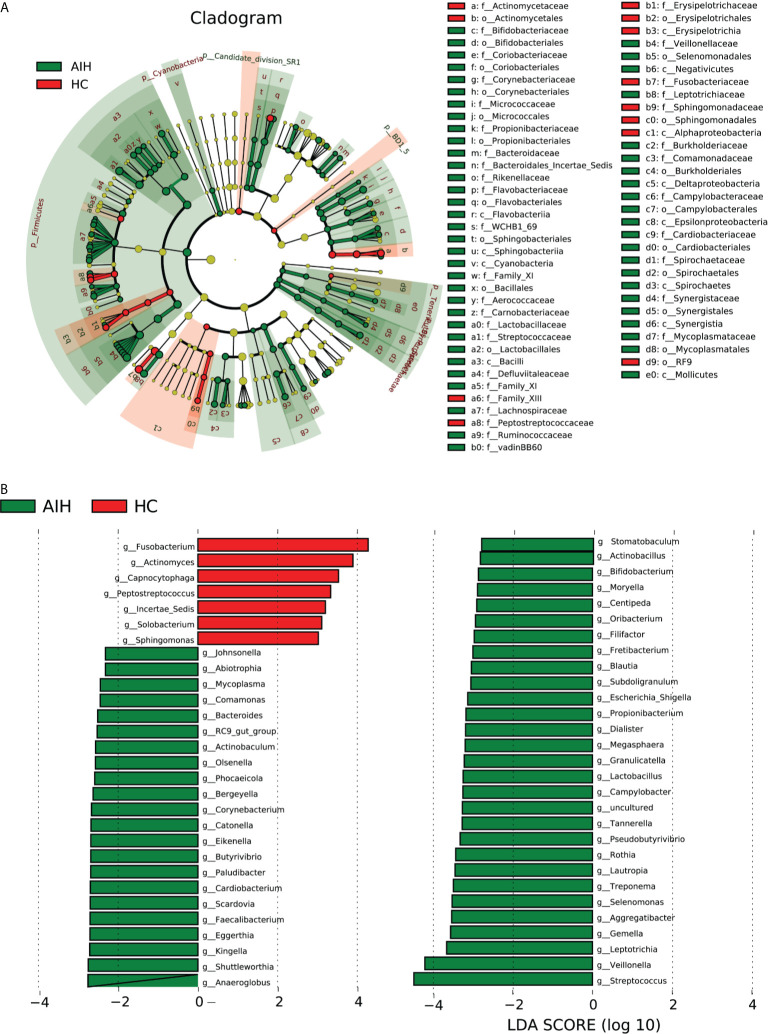
LEfSe and LDA analysis based on OTUs characterize the microbiome between AIH and HCs. **(A)** The phylogenetic tree diagram by the LEfSe method showed the phylogenetic distribution of the oral microbiome related to AIH and HCs. The circles radiating from the inside to the outside represented the classification level from the phylum to the genus. Each circle at levels represented a classification at that level, and the diameter of the circle represents its relative abundance. The uniform coloring with no significant difference was yellow, and the biomarker with significant difference followed the grouping color for coloring. Species with an LDA score greater than 2 and a P value less than 0.05 were considered different species. **(B)** LDA score histogram showed the oral microbiome with significant differences between the two groups. The higher the LDA score, the higher the importance of microbial biomarkers. The default LDA score was greater than 2, and the P-value was less than 0.05, which was considered to indicate differential species. LDA, linear discriminant analysis; LEfSe, linear discriminant analysis effect size; OTUs, operational taxonomic units; AIH, autoimmune hepatitis; HCs, healthy controls.

The cladogram in **Figure 6A** graphically displays the phylogenetic distribution of oral microbiomes associated with AIH and HCs **(** **Supplementary Data S15** **)**. For the selected taxa, the histogram of the LDA scores in **Figure 6** was calculated, which showed the bacteria with significant difference between the AIH and HCs.

Correspondingly, 51 kinds of biomarkers, including *Streptococcus, Veillonella, Leptotrichia, Gemella, Aggregatibacter, Selenomonas* and *Rothia*, were significantly enriched in AIH patients (P<0.05; LDA score>2) and have proven to be microbial dominant genera. Furthermore, the abundances of 7 genera, Fusobacterium, Actinomyces, Capnocytophaga, Peptostreptococcus, Incertae_Sedis, Solobacterium and Sphingomonas, were observed to be lower than those in healthy controls (P<0.05; LDA score>2) (**Supplementary Data S16** and **S17** **)**.

### Gene Function Analysis

**Figure 7:**
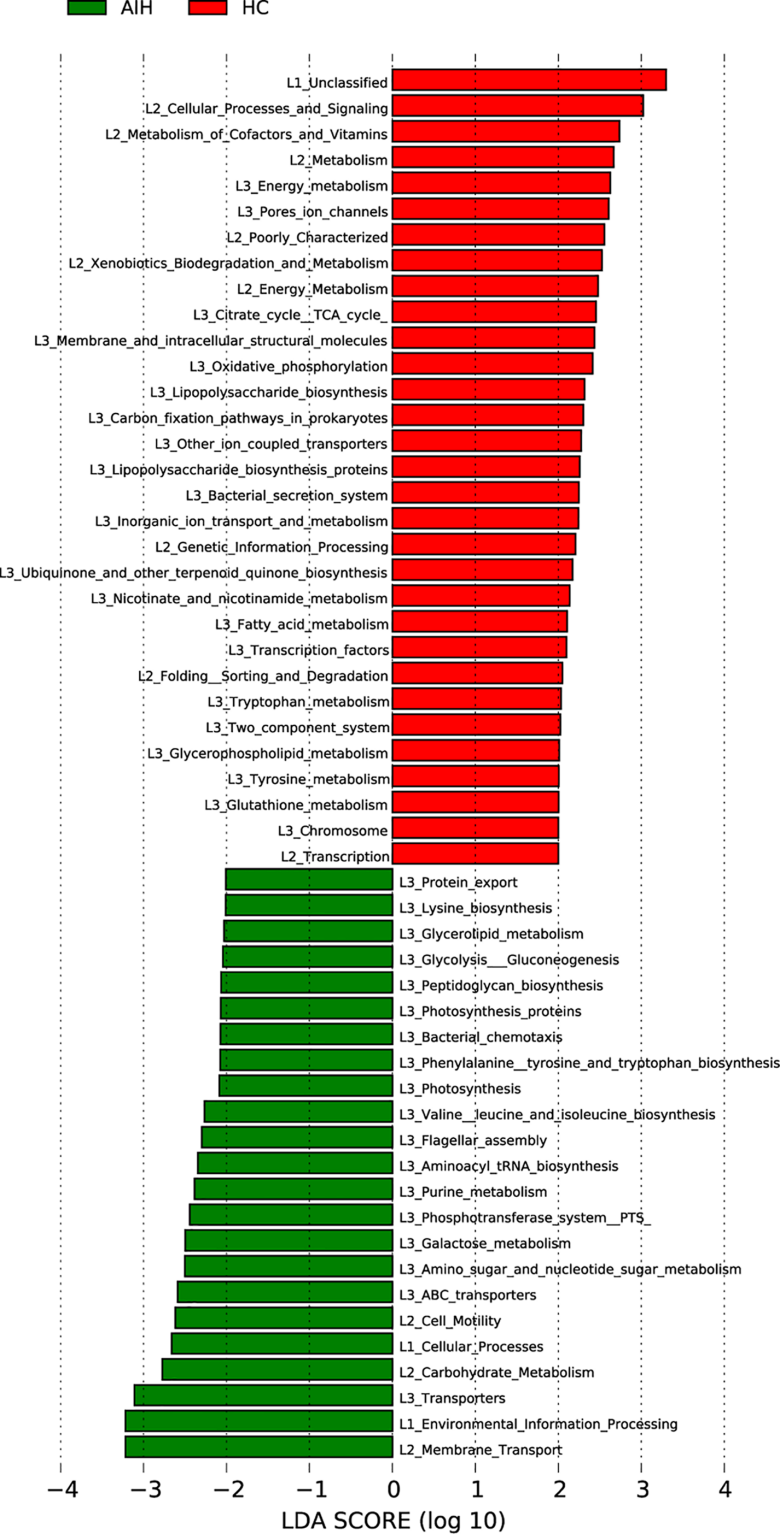
Functional analysis of predicted metagenomes. LEfSe method was used to identify significantly enriched KEGG pathways between AIH and HCs. The higher the LDA score, the higher the importance of microbial biomarkers. The default LDA score was greater than 2, and the P-value was less than 0.05, which was considered as a significantly enriched KEGG pathway. LDA, linear discriminant analysis; LEfSe, linear discriminant analysis effect size AIH, autoimmune hepatitis; HCs, healthy controls.

The cladogram in **Figure 7** graphically displayed the phylogenetic distribution of gene function which was associated with AIH and HCs. As can be found from Lefse LDA score of KEGG direct line homologous gene cluster (KO) annotation, there were 23 kinds of gene function certified to be dominant in AIH group, including Membrane Transport, Environmental Information Processing, Transporters, Carbohydrate Metabolism, Flagellar assembly, Cellular Processes, Cell Motility, Galactose metabolism, Glycolysis Gluconeogenesis and Glycerolipid metabolism, while there were 31 kinds of gene functions observed to increase in HCs group, including Cellular Processes and Signaling, Metabolism of Cofactors and Vitamins, Energy metabolism, Citrate cycle TCA cycle, Lipopolysaccharide biosynthesis, Fatty acid metabolism. More notably, picrust results found that environmental information processing, glucose metabolism, amino acid metabolism, genetic information processing and other functions were changed in the AIH group and HC group accordingly.

### Correlation Analysis Between Oral Microbiomes and Clinical Characteristics

**Figure 8:**
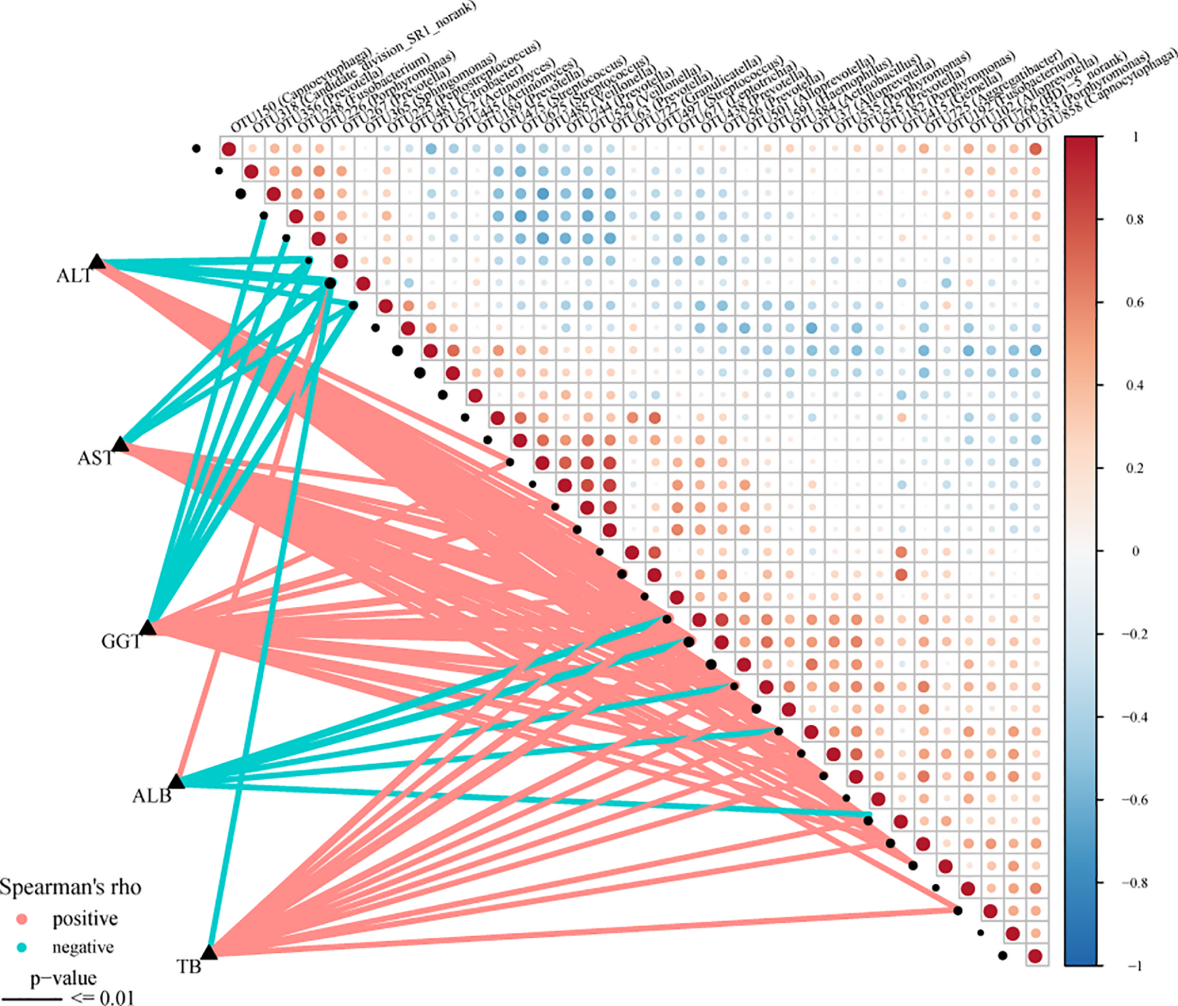
Associations between oral microbiomes and clinical indices of AIH. Distance correlation plots of the relative abundance of 37 OTUand clinical indices, including ALB, GGT, TB, ALT and AST. Positive values (red) indicate positive correlations, and negative values (blue) indicate inverse correlations. The full lines present “P-value ≤ 0.01”. The sign of the correlation was determined using Spearman’s method. Spearman’s method was used to determine the sign of the correlation. AIH, autoimmune hepatitis; ALB, albumin; TB, total bilirubin; GGT, gamma-glutamyltransferase; ALT, alanine aminotransferase; AST, aspartate aminotransferase; AIH, autoimmune hepatitis.

The Spearman rank test was applied to analyze the correlation between the OTU associated with AIH and the clinical characteristics of AIH patients. And we considered possible interfering factors, including age, gender, and BMI. No significant correlation was found among them. The **Figure 8** **(** **Supplementary Data S18** **)** displayed partial Spearman correlation coefficients between 37 OTUs and clinical parameters, including TB, AKP, ALB, ALT, GGT and AST, in the AIH group. The ALB level and the abundance of OTU365 (*Sphingomonas*) were positively correlated (P<0.001, rho=0.383). Correspondingly, the abundances of 5 OTUs, *OTU438* (*Prevotella*) (P<0.001, rho=-0.426), *OTU56* (*Prevotella*) (P<0.001, rho=–0.465), *OTU591* (*Haemophilus*) (P<0.001, rho=-0.366), *OTU37* (*Alloprevotella*) (P<0.001, rho=-0.300) and *OTU415* (*Gemella*) (P<0.001, rho=-0.340) were negative to ALB level. Moreover, the abundances of 3 OTUs, *OTU267* (*Prevotella*) (P<0.001, rho=-0.473), *OTU365* (*Sphingomonas*) (P<0.001, rho=-0.533) and *OTU225* (*Peptostreptococcus*) (P<0.001, rho=-0.348) were negative to ALT level and AST level **(** **Supplementary Data S19** **)**.

### Potential Oral Microbiome-Based Signature in Diagnosis

**Figure 9:**
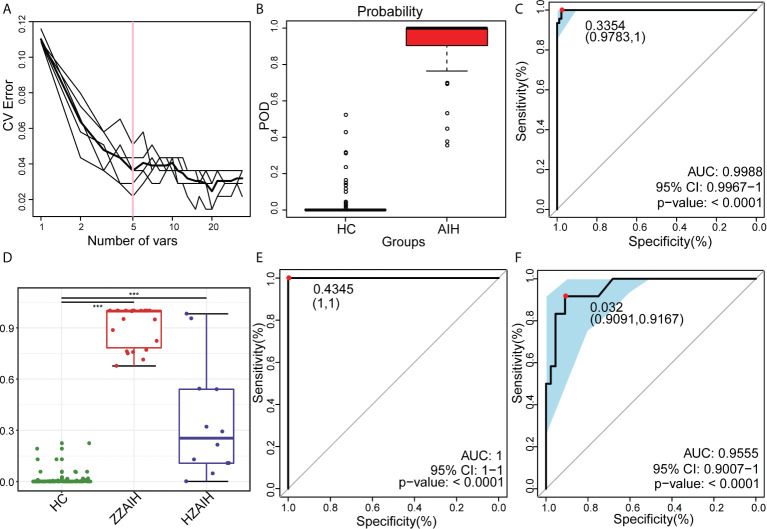
Oral microbiome as non-invasive diagnostic model for AIH. **(A)** As shown in the cross-validation error curve, we used the random forest models to identify five OTUs as the optimal biomarkers. **(B)** The POD value was significantly increased in AIH versus HCs. **(C)** The POD index achieved an AUC value of 99.88% with a 95% CI of 99.67% to 100% between AIH patients and HCs in the discovery phase. **(D)** The POD value of the ZZ-AIH group from Zhengzhou (n=46) and the HZ-AIH group from Hangzhou (n=12) were significantly higher than the POD value of the HCs group (n=44) (P<0.001). **(E)** The POD index achieved an AUC value of 100% between AIH and HCs in the validation phase. **(F)** Twelve AIH patients from Hangzhou were cross-regionally validated with 44 healthy controls. The AUC value reached 95.55%, and its 95% CI was 90.07%-100% (p<0.0001). OTUs, operational taxonomic units; POD, probability of disease; CV error, cross-validation error; AUC, area under the curve; AIH, autoimmune hepatitis; HCs, healthy controls.

In the discovery phase, the least OTUs which could distinguish the differences between the AIH group (N=46) and HC group (N=92) most accurately were found through random forest models. As the cross-validation error curve **(** **Figure 9A** **)** showed, five OTU-based biomarkers, *OTU412 (Selenomonas), OTU277 (Corynebacterium), OTU315 (Leptotrichia), OTU42 (Rothia)* and *OTU484 (Selenomonas)*, were selected as the optimal set with minimum cross-validation error **(** **Supplementary Data S20** **)**.

The probability of disease (POD) index was calculated using the flora data and 37 OTU biomarkers. The previously obtained microflora data and 5 OTU biomarkers were fully utilized to calculate the POD index **(** **Figure 9B** and **Supplementary Data S21**). The POD index was significantly higher in AIH patients compared with HCs. These data showed that the POD index based on OTU markers has great diagnostic potential in AIH.

Of greater significance, when it came to the receiver operating characteristic (ROC) curve for the evaluation of the constructed models, those AIH-associated microbial OTUs distinguished AIH from HCs with an area under the curve (AUC) of approximately 99.88%, and the 95% confidence interval (CI) was 99.67%-100% (P<0.0001), which suggested the strong diagnostic potential of oral microbial markers for AIH **(** **Figure 9C** and **Supplementary Data S22** **)**.

Furthermore, in the validation phase, 44 HCs and 22 AIH patients were used to validate the diagnostic efficacy of the microbial biomarkers for AIH. It is extremely encouraging that the combination of five OTU-based biomarkers achieved an AUC value of 100% between the AIH and HC groups in the validation phase **(** **Figure 9E** and **Supplementary Data S23**). Additionally, 12 AIH patients from Hangzhou were cross-regionally validated with 44 healthy controls. The AUC value reached 95.55%, and its 95% CI was 90.07%-100% (p<0.0001) **(** **Figure 9F** and **Supplementary Data S24**). We also calculated the POD value of each participant from different cohorts and compared the mean POD values of the control and AIH groups at the validation stage and the independent diagnosis stage. The POD values of the ZZ-AIH group from Zhengzhou (n=46) and the HZ-AIH group from Hangzhou (n=12) were significantly higher than the POD value of the HC group (n=44) (P<0.001) **(** **Figure 9D** **).**

## Discussion

This study is the first to report alterations in oral microbiome characteristics in AIH patients from five aspects: species diversity, community composition, gene function, correlation with liver function index and diagnostic model.

In this study, the characteristics of the oral microbiome in AIH patients in Central China were reported for the first time. The oral microbiome of 68 AIH patients and 136 paired HCs from central China were sequenced with 16S rRNA genes. We randomly divided all saliva samples into the discovery cohort (46 AIH and 92 HCs) and validation cohort (22 AIH and 44 HCs). In addition, we collected saliva samples from 12 AIH patients in Hangzhou for cross-regional validation. From the aspects of microbial diversity, microbial community composition, gene function analysis, the correlation between microorganisms and liver function index, and microbial diagnosis model, we described the oral microbiome characteristics of AIH patients.

To our surprise, the oral microbial diversity was significantly increased in AIH patients compared with HCs, which essentially differs from prior studies in the fecal microbiome of AIH patients. By reviewing the literature, we found that in cases of mucosal microbiome such as the mouth (Lu et al., 2017), eye (Shin et al., 2016), and vagina (Freitas et al., 2018), higher diversity was often a sign of disease and inflammation. Interestingly, more often than not, a decreased diversity of the fecal microbiome is often related to disease status, such as liver cirrhosis (LC), hepatocellular carcinoma (HCC) and primary biliary cirrhosis (PBC) (Imhann et al., 2018). It is still unclear what exactly leads to such discrepancy in diversity between oral and gut microbial communities. We previously proposed that the oral microbiome is quite distinct from the gut microbiome; that is, it is not recommended to see the oral microbiome and gut microbiome as a whole.

A recent study by Ren et al. (2019) illustrated that fecal microbial diversity was decreased from HCs to cirrhosis, but it was increased from cirrhosis to early HCC. In addition, the fecal microbial richness was increased in colorectal carcinoma versus adenoma. Thus, greater richness or diversity in the bacterial community might indicate the overgrowth of various harmful bacteria in patients. Moreover, Flemer et al. (2018) detected similar oral microbiota in both the oral cavity and colonic mucosa, including colon tumors patients and children with Crohn’s disease.

In another major study on the salivary microbiome and gut microbiome of controls and cirrhotic patients, Bajaj et al. (2015) reported that fecal and saliva exhibited different microbiomes in both HCs and cirrhotic patients. *Streptococcaceae* was dominant in the salivary microbiome. In contrast, the major family in stool was *Bacteroidaceae*. In addition, compared with the correlation between the salivary microbiome and that of cirrhotic patients, the correlation between the stool microbiome and that of cirrhotic patients appeared to be superior in both strength and importance. In celiac disease and inflammatory bowel disease, the relationship between oral and gut inflammation has also been reported, and changes were similar to those in the gut (Murase et al., 2019).

Different diseases, such as colorectal cancer (CRC), hepatocellular carcinoma (HCC) and inflammatory bowel disease (IBD), were characterized by their unique alteration of microbial composition. In this study, it was proven through NMDS, PCA and PCoA that there was a significant difference in communities of oral microbiome between AIH patients and HCs. Based on the LefSe analysis and LDA score, the oral microbial communities significantly differed between these groups. Seven genera, including *Fusobacterium, Actinomyces, Capnocytophaga, Peptostreptococcus, Incertae_Sedis, Solobacterium* and *Sphingomonas*, were dominant in the HC group, while 51 genera, including *Streptococcus, Veillonella, Leptotrichia, Gemella, Aggregatibacter, Selenomonas* and *Rothia*, were enriched in the AIH group and were considered microbial dominant genera.

According to a fivefold cross-validation on a random forest model, five OTU biomarkers, OTU412 (*Selenomonas*), OTU277 (*Corynebacterium*), OTU315 (*Leptotrichia*), OTU42 (*Rothia*) and OTU484 (*Selenomonas*), achieved an AUC of 99.88% between 46 AIH and 92 HC samples and an AUC of 95.55% in cross-regional validation between 12 AIH patients and 44 HCs. It is now possible to state that there is an obvious alteration in the oral microbiome of AIH patients, which therefore assists substantially in the diagnosis of AIH, especially for atypical cases.

We further investigated the association between the oral microbiome and clinical parameters. There were positive correlations between ALB level and the abundance of OTU365 (Sphingomonas). Correspondingly, the abundances of 5 OTUs, *OTU438 (Prevotella)*, *OTU56 (Prevotella)*, *OTU591 (Haemophilus)*, *OTU37 (Alloprevotella)* and *OTU415 (Gemella)* were negative to ALB level. Moreover, the abundances of 3 OTUs, OTU267 *(Prevotella)*, *OTU365 (Sphingomonas)* and *OTU225 (Peptostreptococcus)* were negative to ALT level and AST level. These findings remind us of the potential association between oral microbiomes and AIH severity.

Notably, the results from PICRUSt consistently revealed pronounced alterations in several gene functions. We found 23 gene functions, including Membrane Transport, Environmental Information Processing, Transporters, Carbohydrate Metabolism, Flagellar assembly, Cellular Processes, Cell Motility, Galactose metabolism, Glycolysis Gluconeogenesis and Glycerolipid metabolism, dominant in AIH and 31 gene functions prevailing in HCs, including Cellular Processes and Signaling, Metabolism of Cofactors and Vitamins, Energy metabolism, Citrate cycle TCA cycle, Lipopolysaccharide biosynthesis, Fatty acid metabolism. The main feature of AIH is inflammation of the liver interface. More energy was needed to meet the consumption of the immune system as the disease progresses. The immune system competes with other programs in the body for energy. In addition, the energy supply is significantly disordered in the body, which is mainly manifested in the large consumption of nutrients (such as carbohydrate, proteins and lipids) and the enhancement of degradation in carbohydrate, lipid and protein (Freitas et al., 2018; Li et al., 2021). In our study, the gene function which was significant enriched in AIH were mainly associated with energy metabolism. These alterations in gene functions convincingly show the potential influence of oral microbiomes on metabolism. The oral microbiomes of AIH patients are likely to contribute to the development of the disease by influencing metabolic pathways (Wang et al., 2016; Balitzer et al., 2017).

Furthermore, the participants of our study were outpatients of AIH, who were in the relatively early stage of AIH and had only mild symptoms. Participants who consumed UDCA and steroids were excluded because of the potential changes in the oral microbiome induced by drugs. Therefore, early diagnosis and non-invasive diagnosis could be realized by the microbial diagnosis model for AIH patients. So, this study has important clinical application value in clinical diagnosis and treatment.

Finally, the results of human and animal experiments have shown that the development of autoimmune diseases is related to the homeostasis of microbial communities. The microbiome can play a positive role in healthy people in a variety of ways: protecting the body from pathogenic microorganisms; producing energy sources for intestinal epidermal cells and anti-inflammatory factors; synthesizing necessary vitamins and amino acids; and affecting the development of the immune system (Routy et al., 2018). In turn, the composition and colonization of microbiome communities are also affected by the human body, including neonatal production, breastfeeding, diet and antibiotic treatment. Once the microbiome is out of order, the functions listed above cannot be maintained, and the development of autoimmune diseases is promoted as well (Sanna et al., 2019).

Having attempted to draw a fine distinction in oral microbiome between AIH and HCs, perhaps the most serious shortcoming of this article was that individual’s microbial characteristics may be altered by many factors: time (the type of community in the oral cavity were more unstable during the sampling period), age, diet, extreme environment and antibiotic treatment (Li et al., 2016). We expand the sample size and improve the sampling procedures to minimize the impact of other interference factors (Balitzer et al., 2017; Gopalakrishnan et al., 2018). There were other limitations in this study including the LEfSe method and the PICRUSt method. LEfSe is a common tool used to find species with significant differences between groups in microbial research. The result of LEfSe can not be adjusted for FDR, leading to potential errors to some extent. However, it ​has been widely recognized in the current field (Cabral et al., 2019; Claria et al., 2019). In addition, we admit that PICRUSt cannot replace a metagenomic analysis to a large extent. However, PICRUSt is the first tool developed to predict the function of microbial communities based on the 16S rRNA gene sequence and has been widely recognized in the microbial field (Langille et al., 2013; Li et al., 2017). Another limitation was that this study illuminated the characteristics of oral microbiome in AIH, and it really cannot prove the causality between AIH and oral microbiome. At present, in the research field of microbiome, animal experiments were needed to validate the causality between microbiome and diseases. So far, studies in obesity have successfully clarified the causal relationship between obesity and microbiome (Zhao, 2013). These were the limitations that need to be improved in our future research.

## Conclusion

This study is the first to characterize the oral microbiome in AIH patients and to report the successful establishment of a diagnostic model and the cross-regional validation of microbial markers for AIH. Importantly, oral microbiota-targeted biomarkers may be able to serve as powerful and noninvasive diagnostic tools for AIH.

## Data Availability Statement

The datasets presented in this study can be found in online repositories. The names of the repository/repositories and accession number(s) can be found in the article/**Supplementary Material**.

## Ethics Statement

The studies involving human participants were reviewed and approved by the institutional review Board of the First affiliated Hospital of Zhengzhou University. The patients/participants provided their written informed consent to participate in this study.

## Author Contributions

ZY and ZR designed the study. BR, JML, HFL, HXL, JL, HQZ, YS and YZ collected clinical samples and performed the experiments. YF, ZW and YJ analyzed the data. BR, JML, ZR and ZY wrote the manuscript. All authors contributed to the article and approved the submitted version.

## Funding

The study was supported by the China Postdoctoral Science Foundation (2020T130609 and 2020T130109ZX), Henan Province Science and Technology Project (202102310055 to ZR), National Key Research and Development Program of China (2018YFC2000500), National Natural Science Foundation of China (U2004121), Key Scientific Research Projects of Higher Education Institutions in Henan Province (20A320056), and the opening foundation of the State Key Laboratory for Diagnosis and Treatment of Infectious Diseases and Collaborative Innovation Center for Diagnosis and Treatment of Infectious Diseases, The First Affiliated Hospital, College of Medicine, Zhejiang University (SKLID2019KF03).

## Conflict of Interest

The authors declare that the research was conducted in the absence of any commercial or financial relationships that could be construed as a potential conflict of interest.

## References

[B1] Abe K. Takahashi A. Fujita M. Imaizumi H. Hayashi M. Okai K.. (2018). Dysbiosis of Oral Microbiota and its Association With Salivary Immunological Biomarkers in Autoimmune Liver Disease. PloS One 13 (7), e0198757. 10.1371/journal.pone.0198757 29969462 PMC6029758

[B2] Aguayo S. Schuh C. M. A. P. Vicente B. Aguayo L. G. (2018). Association Between Alzheimer’s Disease and Oral and Gut Microbiota: Are Pore Forming Proteins the Missing Link? J. Alzheimers Dis. 65 (1), 29–46. 10.3233/JAD-180319 30040725

[B3] Akcali A. Bostanci N. Ozcaka O. Ozturk-Ceyhan B. Gumus P. Buduneli N.. (2014). Association Between Polycystic Ovary Syndrome, Oral Microbiota and Systemic Antibody Responses. PloS One 9 (9), e108074. 10.1371/journal.pone.0108074 25232962 PMC4169459

[B4] Anand L. Choudhury A. Bihari C. Sharma B. C. Kumar M. Maiwall R.. (2019). Flare of Autoimmune Hepatitis Causing Acute on Chronic Liver Failure: Diagnosis and Response to Corticosteroid Therapy. Hepatology 70 (2), 587–596. 10.1002/hep.30205 30113706

[B5] Bajaj J. S. Betrapally N. S. Hylemon P. B. Heuman D. M. Daita K. White M. B.. (2015). Salivary Microbiota Reflects Changes in Gut Microbiota in Cirrhosis With Hepatic Encephalopathy. Hepatology 62 (4), 1260–1271. 10.1002/hep.27819 25820757 PMC4587995

[B6] Balitzer D. Shafizadeh N. Peters M. G. Ferrell L. D. Alshak N. Kakar S. (2017). Autoimmune Hepatitis: Review of Histologic Features Included in the Simplified Criteria Proposed by the International Autoimmune Hepatitis Group and Proposal for New Histologic Criteria. Mod. Pathol. 30 (5), 773–783. 10.1038/modpathol.2016.267 28106105

[B7] Cabral D. J. Penumutchu S. Reinhart E. M. Zhang C. Korry B. J. Wurster J. I.. (2019). Microbial Metabolism Modulates Antibiotic Susceptibility Within the Murine Gut Microbiome. Cell Metab. 30 (4), 800–823.e807. 10.1016/j.cmet.2019.08.020 31523007 PMC6948150

[B8] Caussy C. Tripathi A. Humphrey G. Bassirian S. Singh S. Faulkner C.. (2019). A Gut Microbiome Signature for Cirrhosis Due to Nonalcoholic Fatty Liver Disease. Nat. Commun. 10 (1), 1406. 10.1038/s41467-019-09455-9 30926798 PMC6440960

[B9] Claria J. Moreau R. Fenaille F. Amoros A. Junot C. Gronbaek H.. (2019). Orchestration of Tryptophan-Kynurenine Pathway, Acute Decompensation, and Acute-on-Chronic Liver Failure in Cirrhosis. Hepatology 69 (4), 1686–1701. 10.1002/hep.30363 30521097

[B10] Corwin E. J. Hogue C. J. Pearce B. Hill C. C. Read T. D. Mulle J.. (2017). Correction to: Protocol for the Emory University African American Vaginal, Oral, and Gut Microbiome in Pregnancy Cohort Study. BMC Pregnancy Childbirth 17 (1), 395. 10.1186/s12884-017-1550-y 29179694 PMC5704355

[B11] Deschasaux M. Bouter K. E. Prodan A. Levin E. Groen A. K. Herrema H.. (2018). Depicting the Composition of Gut Microbiota in a Population With Varied Ethnic Origins But Shared Geography. Nat. Med. 24 (10), 1526–1531. 10.1038/s41591-018-0160-1 30150717

[B12] Edgar R. C. (2013). UPARSE: Highly Accurate OTU Sequences From Microbial Amplicon Reads. Nat. Methods 10 (10), 996–998. 10.1038/nmeth.2604 23955772

[B13] Edgar R. C. Haas B. J. Clemente J. C. Quince C. Knight R. (2011). UCHIME Improves Sensitivity and Speed of Chimera Detection. Bioinformatics 27 (16), 2194–2200. 10.1093/bioinformatics/btr381 21700674 PMC3150044

[B14] Flemer B. Warren R. D. Barrett M. P. Cisek K. Das A. Jeffery I. B.. (2018). The Oral Microbiota in Colorectal Cancer is Distinctive and Predictive. Gut 67 (8), 1454–1463. 10.1136/gutjnl-2017-314814 28988196 PMC6204958

[B15] Fouhy F. Watkins C. Hill C. J. O’Shea C. A. Nagle B. Dempsey E. M.. (2019). Perinatal Factors Affect the Gut Microbiota Up to Four Years After Birth. Nat. Commun. 10 (1), 1517. 10.1038/s41467-019-09252-4 30944304 PMC6447568

[B16] Freitas A. C. Bocking A. Hill J. E. Money D. M. Group, Vogue Research (2018). Increased Richness and Diversity of the Vaginal Microbiota and Spontaneous Preterm Birth. Microbiome 6 (1), 117. 10.1186/s40168-018-0502-8 29954448 PMC6022438

[B17] Gao L. Xu T. Huang G. Jiang S. Gu Y. Chen F. (2018). Oral Microbiomes: More and More Importance in Oral Cavity and Whole Body. Protein Cell 9 (5), 488–500. 10.1007/s13238-018-0548-1 29736705 PMC5960472

[B18] Gopalakrishnan V. Helmink B. A. Spencer C. N. Reuben A. Wargo J. A. (2018). The Influence of the Gut Microbiome on Cancer, Immunity, and Cancer Immunotherapy. Cancer Cell 33 (4), 570–580. 10.1016/j.ccell.2018.03.015 29634945 PMC6529202

[B19] Imhann F. Vich Vila A. Bonder M. J. Fu J. Gevers D. Visschedijk M. C.. (2018). Interplay of Host Genetics and Gut Microbiota Underlying the Onset and Clinical Presentation of Inflammatory Bowel Disease. Gut 67 (1), 108–119. 10.1136/gutjnl-2016-312135 27802154 PMC5699972

[B20] Kakiyama G. Pandak W. M. Gillevet P. M. Hylemon P. B. Heuman D. M. Daita K.. (2013). Modulation of the Fecal Bile Acid Profile by Gut Microbiota in Cirrhosis. J. Hepatol. 58 (5), 949–955. 10.1016/j.jhep.2013.01.003 23333527 PMC3936319

[B21] Koren O. Spor A. Felin J. Fak F. Stombaugh J. Tremaroli V.. (2011). Human Oral, Gut, and Plaque Microbiota in Patients With Atherosclerosis. Proc. Natl. Acad. Sci. U.S.A. 108 (Suppl 1), 4592–4598. 10.1073/pnas.1011383107 20937873 PMC3063583

[B22] Langille M. G. Zaneveld J. Caporaso J. G. McDonald D. Knights D. Reyes J. A.. (2013). Predictive Functional Profiling of Microbial Communities Using 16S rRNA Marker Gene Sequences. Nat. Biotechnol. 31 (9), 814–821. 10.1038/nbt.2676 23975157 PMC3819121

[B23] Lapidot Y. Amir A. Ben-Simon S. Veitsman E. Cohen-Ezra O. Davidov Y.. (2021). Alterations of the Salivary and Fecal Microbiome in Patients With Primary Sclerosing Cholangitis. Hepatol. Int. 15 (1), 191–201. 10.1007/s12072-020-10089-z 32949377

[B24] Ling Z. Liu X. Cheng Y. Jiang X. Jiang H. Wang Y.. (2015). Decreased Diversity of the Oral Microbiota of Patients With Hepatitis B Virus-Induced Chronic Liver Disease: A Pilot Project. Sci. Rep. 5, 17098. 10.1038/srep17098 26606973 PMC4660595

[B25] Li S. S. Niu M. Jing J. Huang Y. Zhang Z. T. Chen S. S.. (2021). Metabolomic Signatures of Autoimmune Hepatitis in the Development of Cirrhosis. Front. Med. (Lausanne) 8, 644376. 10.3389/fmed.2021.644376 33777984 PMC7994277

[B26] Li D. Wang P. Wang P. Hu X. Chen F. (2016). The Gut Microbiota: A Treasure for Human Health. Biotechnol. Adv. 34 (7), 1210–1224. 10.1016/j.biotechadv.2016.08.003 27592384

[B27] Li G. Xie C. Lu S. Nichols R. G. Tian Y. Li L.. (2017). Intermittent Fasting Promotes White Adipose Browning and Decreases Obesity by Shaping the Gut Microbiota. Cell Metab. 26 (4), 672–685.e674. 10.1016/j.cmet.2017.08.019 28918936 PMC5668683

[B28] Lou J. Jiang Y. Rao B. Li A. Ding S. Yan H.. (2020). Fecal Microbiomes Distinguish Patients With Autoimmune Hepatitis From Healthy Individuals. Front. Cell Infect. Microbiol. 10, 342. 10.3389/fcimb.2020.00342 32850468 PMC7416601

[B29] Lu H. F. Li A. Zhang T. Ren Z. G. He K. X. Zhang H.. (2017). Disordered Oropharyngeal Microbial Communities in H7N9 Patients With or Without Secondary Bacterial Lung Infection. Emerg. Microbes Infect. 6 (12), e112. 10.1038/emi.2017.101 29259328 PMC5750457

[B30] Lu H. Ren Z. Li A. Li J. Xu S. Zhang H.. (2019). Tongue Coating Microbiome Data Distinguish Patients With Pancreatic Head Cancer From Healthy Controls. J. Oral. Microbiol. 11 (1), 1563409. 10.1080/20002297.2018.1563409 30728915 PMC6352935

[B31] Lu H. Ren Z. Li A. Zhang H. Jiang J. Xu S.. (2016). Deep Sequencing Reveals Microbiota Dysbiosis of Tongue Coat in Patients With Liver Carcinoma. Sci. Rep. 6, 33142. 10.1038/srep33142 27605161 PMC5015078

[B32] Lu H. Wu Z. Xu W. Yang J. Chen Y. Li L. (2011). Intestinal Microbiota was Assessed in Cirrhotic Patients With Hepatitis B Virus Infection. Intestinal Microbiota of HBV Cirrhotic Patients. Microb. Ecol. 61 (3), 693–703. 10.1007/s00248-010-9801-8 21286703

[B33] Magoc T. Salzberg S. L. (2011). FLASH: Fast Length Adjustment of Short Reads to Improve Genome Assemblies. Bioinformatics 27 (21), 2957–2963. 10.1093/bioinformatics/btr507 21903629 PMC3198573

[B34] Murase K. Watanabe T. Arai S. Kim H. Tohya M. Ishida-Kuroki K.. (2019). Characterization of Pig Saliva as the Major Natural Habitat of Streptococcus Suis by Analyzing Oral, Fecal, Vaginal, and Environmental Microbiota. PloS One 14 (4), e0215983. 10.1371/journal.pone.0215983 31017953 PMC6481863

[B35] Ren Z. Cui G. Lu H. Chen X. Jiang J. Liu H.. (2013). Liver Ischemic Preconditioning (IPC) Improves Intestinal Microbiota Following Liver Transplantation in Rats Through 16s rDNA-based Analysis of Microbial Structure Shift. PloS One 8 (10), e75950. 10.1371/journal.pone.0075950 24098410 PMC3788797

[B36] Ren Z. Jiang J. Lu H. Chen X. He Y. Zhang H.. (2014). Intestinal Microbial Variation may Predict Early Acute Rejection After Liver Transplantation in Rats. Transplantation 98 (8), 844–852. 10.1097/TP.0000000000000334 25321166 PMC4206351

[B37] Ren Z. Jiang J. Xie H. Li A. Lu H. Xu S.. (2017). Gut Microbial Profile Analysis by MiSeq Sequencing of Pancreatic Carcinoma Patients in China. Oncotarget 8 (56), 95176–95191. 10.18632/oncotarget.18820 29221120 PMC5707014

[B38] Ren Z. Li A. Jiang J. Zhou L. Yu Z. Lu H.. (2019). Gut Microbiome Analysis as a Tool Towards Targeted non-Invasive Biomarkers for Early Hepatocellular Carcinoma. Gut 68 (6), 1014–1023. 10.1136/gutjnl-2017-315084 30045880 PMC6580753

[B39] Ren Z. Li Y. Liu J. Li H. Li A. Hong L.. (2018). Coreopsis Tinctoria Modulates Lipid Metabolism by Decreasing Low-Density Lipoprotein and Improving Gut Microbiota. Cell Physiol. Biochem. 48 (3), 1060–1074. 10.1159/000491973 30041165

[B40] Routy B. Le Chatelier E. Derosa L. Duong C. P. M. Alou M. T. Daillere R.. (2018). Gut Microbiome Influences Efficacy of PD-1-based Immunotherapy Against Epithelial Tumors. Science 359 (6371), 91–97. 10.1126/science.aan3706 29097494

[B41] Said H. S. Suda W. Nakagome S. Chinen H. Oshima K. Kim S.. (2014). Dysbiosis of Salivary Microbiota in Inflammatory Bowel Disease and its Association With Oral Immunological Biomarkers. DNA Res. 21 (1), 15–25. 10.1093/dnares/dst037 24013298 PMC3925391

[B42] Sanna S. van Zuydam N. R. Mahajan A. Kurilshikov A. Vich Vila A. Vosa U.. (2019). Causal Relationships Among the Gut Microbiome, Short-Chain Fatty Acids and Metabolic Diseases. Nat. Genet. 51 (4), 600–605. 10.1038/s41588-019-0350-x 30778224 PMC6441384

[B43] Sebode M. Weiler-Normann C. Liwinski T. Schramm C. (2018). Autoantibodies in Autoimmune Liver Disease-Clinical and Diagnostic Relevance. Front. Immunol. 9, 609. 10.3389/fimmu.2018.00609 29636752 PMC5880919

[B44] Shin H. Price K. Albert L. Dodick J. Park L. Dominguez-Bello M. G. (2016). Changes in the Eye Microbiota Associated With Contact Lens Wearing. mBio 7 (2), e00198. 10.1128/mBio.00198-16 27006462 PMC4817251

[B45] Tilg H. Adolph T. E. Gerner R. R. Moschen A. R. (2018). The Intestinal Microbiota in Colorectal Cancer. Cancer Cell 33 (6), 954–964. 10.1016/j.ccell.2018.03.004 29657127

[B46] Wang Y. Wiesnoski D. H. Helmink B. A. Gopalakrishnan V. Choi K. DuPont H. L.. (2018). Fecal Microbiota Transplantation for Refractory Immune Checkpoint Inhibitor-Associated Colitis. Nat. Med. 24 (12), 1804–1808. 10.1038/s41591-018-0238-9 30420754 PMC6322556

[B47] Wang Q. Yang F. Miao Q. Krawitt E. L. Gershwin M. E. Ma X. (2016). The Clinical Phenotypes of Autoimmune Hepatitis: A Comprehensive Review. J. Autoimmun. 66, 98–107. 10.1016/j.jaut.2015.10.006 26614611

[B48] Wei Y. Li Y. Yan L. Sun C. Miao Q. Wang Q.. (2020). Alterations of Gut Microbiome in Autoimmune Hepatitis. Gut 69 (3), 569–577. 10.1136/gutjnl-2018-317836 31201284

[B49] Xiao E. Mattos M. Vieira G. H. A. Chen S. Correa J. D. Wu Y.. (2017). Diabetes Enhances IL-17 Expression and Alters the Oral Microbiome to Increase its Pathogenicity. Cell Host Microbe 22 (1), 120–128.e124. 10.1016/j.chom.2017.06.014 28704648 PMC5701758

[B50] Zhang X. Zhang D. Jia H. Feng Q. Wang D. Liang D.. (2015). The Oral and Gut Microbiomes are Perturbed in Rheumatoid Arthritis and Partly Normalized After Treatment. Nat. Med. 21 (8), 895–905. 10.1038/nm.3914 26214836

[B51] Zhao L. (2013). The Gut Microbiota and Obesity: From Correlation to Causality. Nat. Rev. Microbiol. 11 (9), 639–647. 10.1038/nrmicro3089 23912213

