# Supplementary material for: Oral Microbiome Characteristics in Patients With Autoimmune Hepatitis
Source: Front Cell Infect Microbiol. 2021 May 19;11:656674. doi: 10.3389/fcimb.2021.656674 (PMC8170700; doi:10.3389/fcimb.2021.656674)
Supplement: Supplementary file 1 [file DataSheet_1.docx]

**Supplementary** **Information**

Oral microbiome characteristics in patients with autoimmune hepatitis

Benchen Rao ^1,6†^, Jiamin Lou ^2†^, Haifeng Lu ^3^, Hongxia Liang ^1,6^, Juan Li ^1,6^, Heqi Zhou ^1,6^, Yajuan Fan ^4^, Hua Zhang ^3^, Ying Sun ^1,6^, Yawen Zou ^1,6^, Zhongwen Wu ^3^, Yan Jiang ^5^, Zhigang Ren ^1,6*^, Zujiang Yu ^1,6*^

^1^ Department of Infectious Diseases, the First Affiliated Hospital of Zhengzhou University, Zhengzhou 450052, China;

^2^ Department of Infectious Diseases, Yiwu Central Hospital, Yiwu 322000, Zhejiang Province, China;

^3^ State Key Laboratory for Diagnosis and Treatment of Infectious Disease, the First Affiliated Hospital, School of Medicine, Zhejiang University, Hangzhou 310003, China;

^4^ Department of Nephrology, the First Affiliated Hospital of Zhengzhou University, Zhengzhou 450052, China.

^5^ Department of Neurology, the First Affiliated Hospital of Zhengzhou University, Zhengzhou 450052, China.

^6^ Gene Hospital of Henan Province; Precision Medicine Center, the First Affiliated Hospital of Zhengzhou University, Zhengzhou 450052, China;

†These authors contributed equally to this work.

***Corresponding to**

Zhigang Ren, Ph.D., M.D., Department of Infectious Diseases, the First Affiliated Hospital of Zhengzhou University, 1#, Jianshe East Road, Zhengzhou 450052, China. E-mail: [fccrenzg@zzu.edu.cn](mailto:fccrenzg@zzu.edu.cn)

Zujiang Yu, Ph.D., M.D., Department of Infectious Diseases, the First Affiliated Hospital of Zhengzhou University, 1#, Jianshe East Road, Zhengzhou 450052, China. E-mail: [johnyuem@zzu.edu.cn](mailto:johnyuem@zzu.edu.cn)

**Supplementary Information**

1. **Supplementary figures S1-9:**

**Supplementary figure 1 4**

**Supplementary figure 2 5**

**Supplementary figure 3 6**

**Supplementary figure 4 7**

**Supplementary figure 5 8**

**Supplementary figure 6 9**

**Supplementary figure 7 10**

**Supplementary figure 8 11**

**Supplementary figure 9 12**

**Supplementary Figure 1**

**
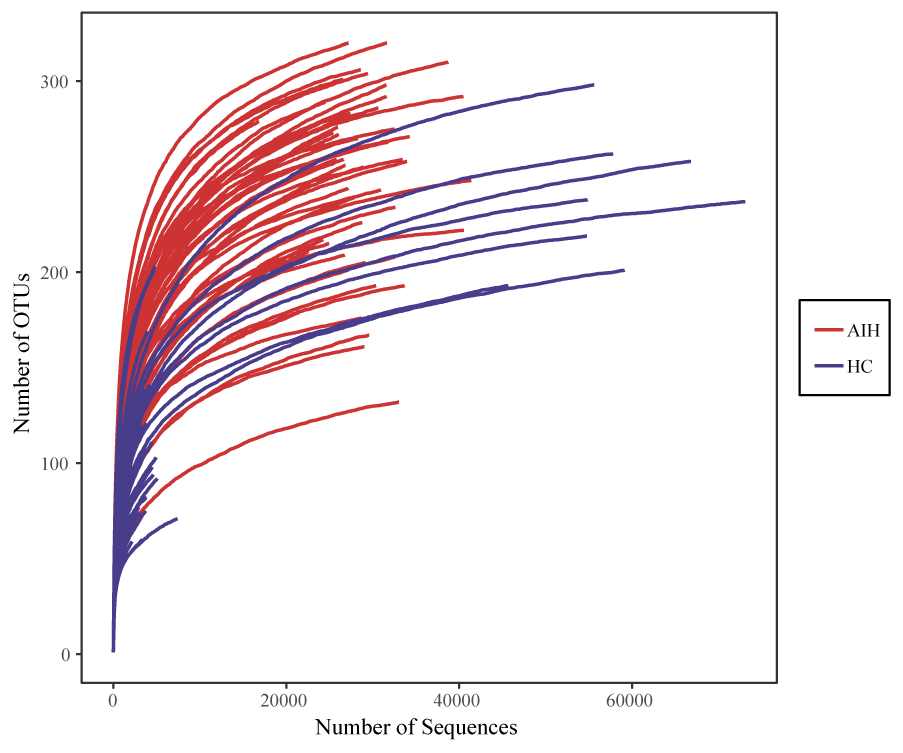
**

**Figure S1. Rarefaction curve of bacterial 16S rRNA gene sequences.** Rarefaction curve can be used to compare the abundance of species in different samples sequenced. For a sample, when the curve tends to be flat, it indicates that the amount of sequencing data is reasonable. AIH, Autoimmune hepatitis; HC, healthy controls; OTUs, Operational Taxonomy Units.

**Supplementary Figure 2**

**
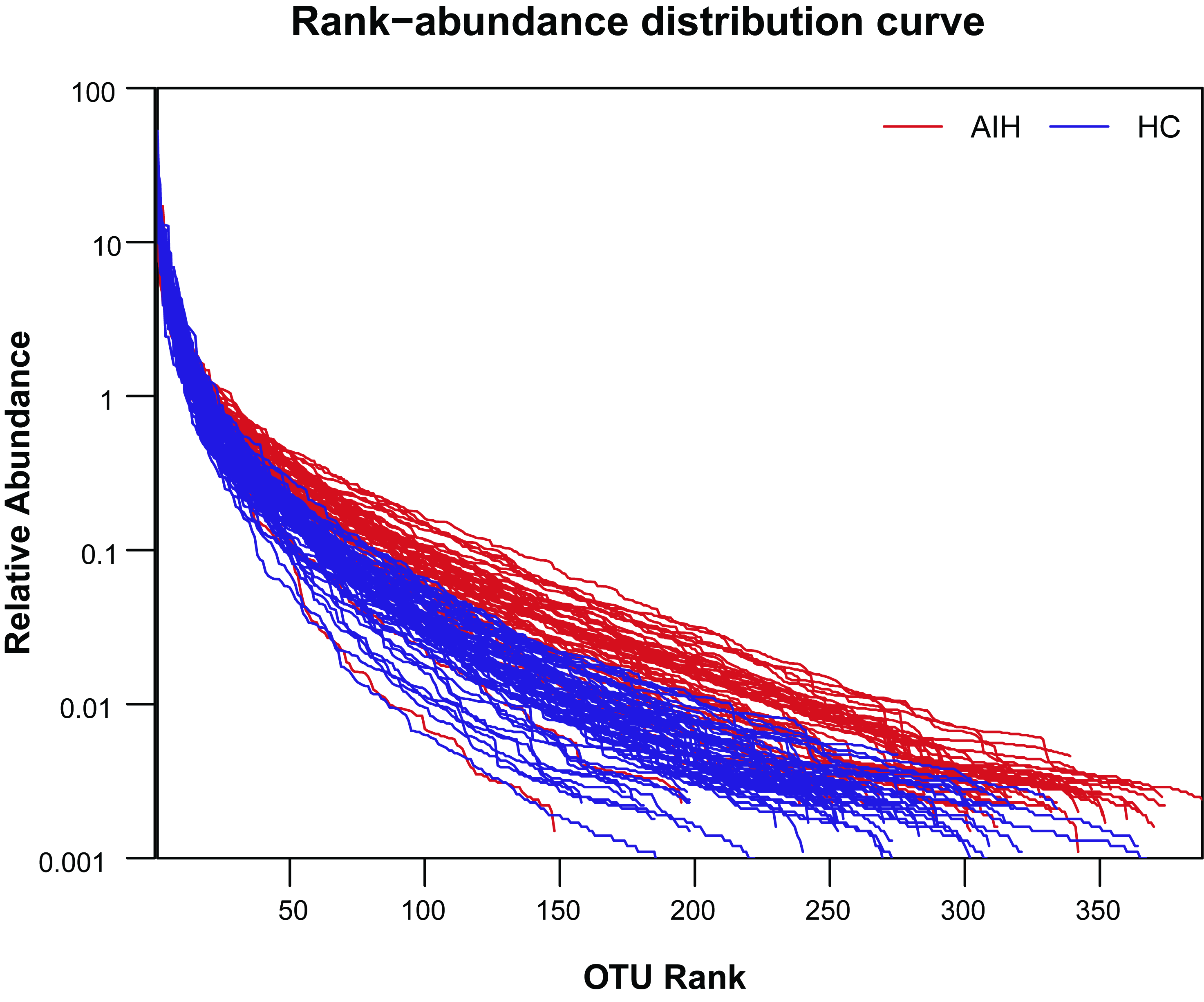
**

**Figure S2.** **Rank-Abundance distribution curve of bacterial 16S rRNA gene sequences.** Rank-Abundance curve showed that the sequencing data of the sample is of high quality. In the horizontal direction, the abundance of the species is reflected by the width of the curve, the larger the range of the curve on the transverse axis is, the higher the abundance of the species is. The shape (smoothness) of the curve reflects the uniformity of the species in the sample, and the flatter the curve is, the more uniform the species distribution is. AIH, Autoimmune hepatitis; HC, healthy controls; OTUs, Operational Taxonomy Units.

**Supplementary Figure 3
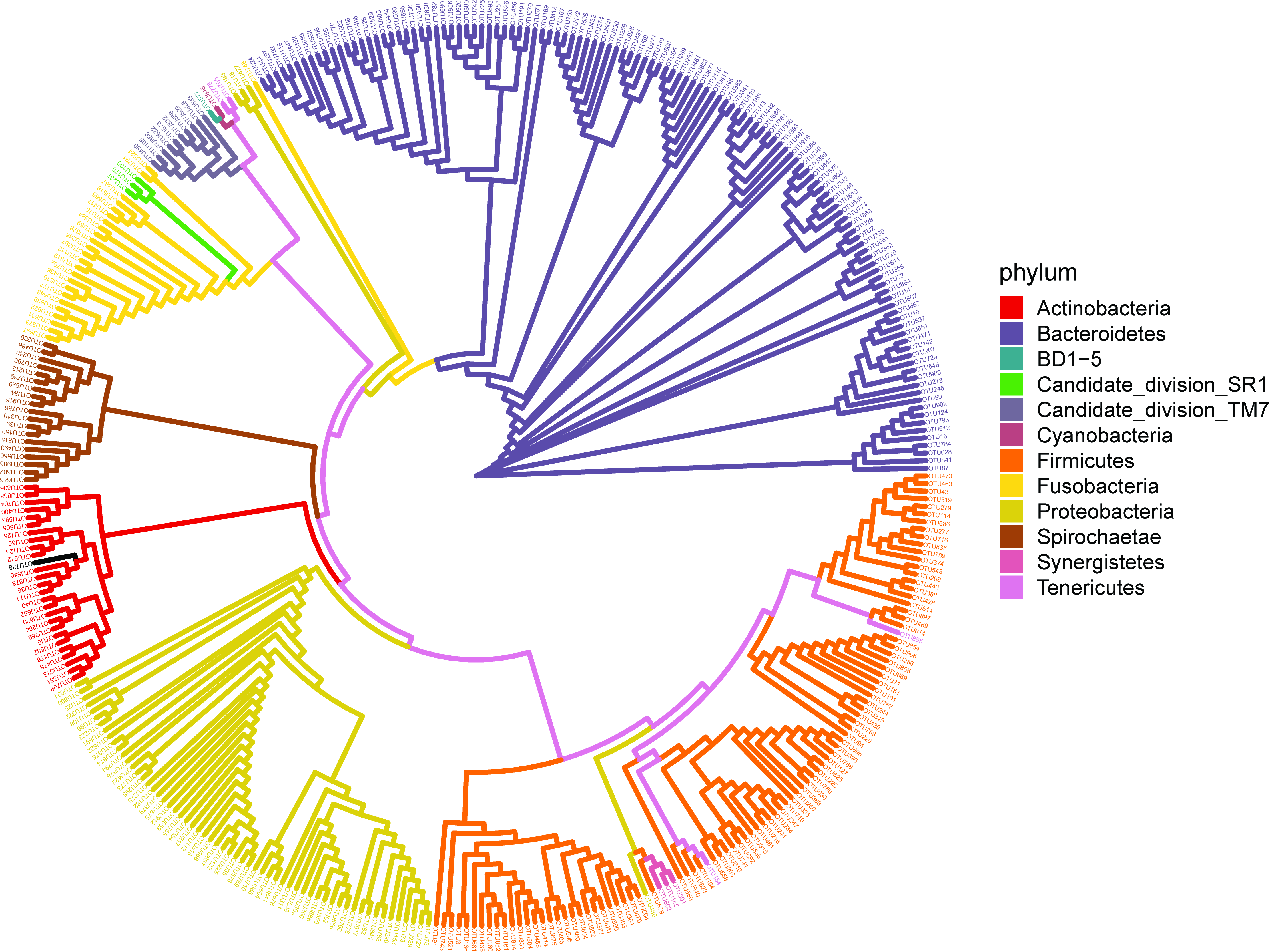
**

**Figure S3.** **Phylogenetic tree of OTUs phylum.** The phylogenetic tree of OTUs phylum described the systematic evolution relationship of 12 phyla. Each branch in phylogenetic tree represents a phylum, and the length of the branch is the evolutionary distance between the two phyla, that is, the degree of difference of the phyla. More information concerned with phylogenetic tree can be seen in Supplementary data S6. OTUs, Operational Taxonomy Units.

**Supplementary Figure 4**

**
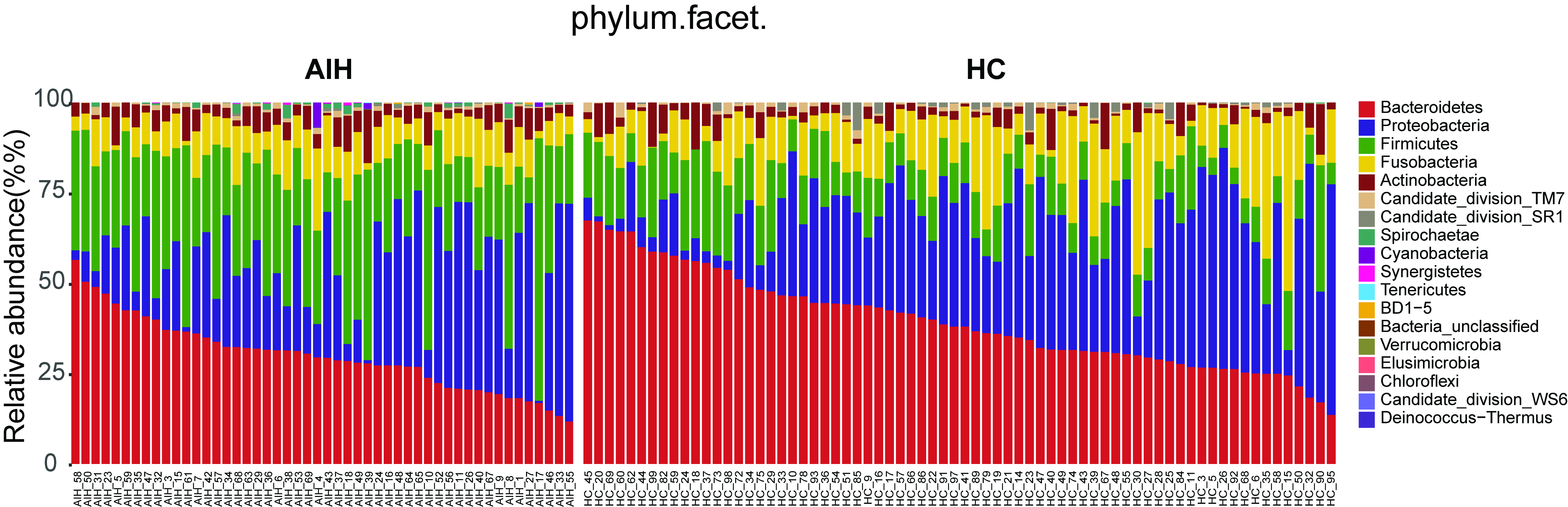
**

**Figure S4. Composition of oral microbiota** **at the phylum level between AIH and HCs.** The microbial community barplot of AIH and HC visually reflected the relative abundance of microbiome at phylum level for each sample. AIH, Autoimmune hepatitis; HCs, healthy controls.

**Supplementary Figure 5**


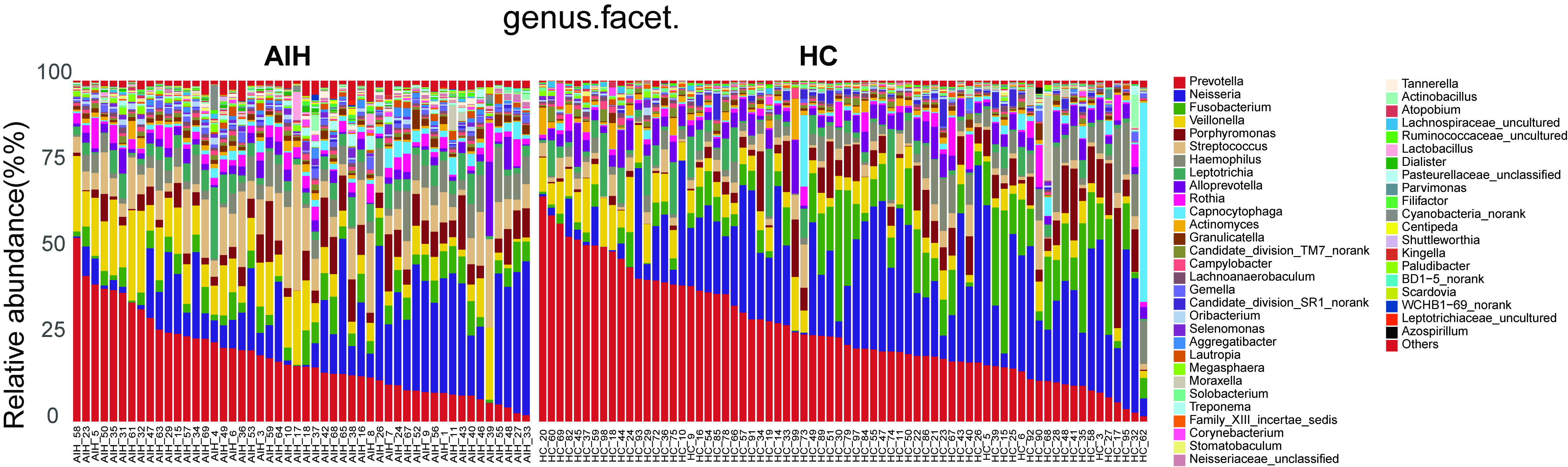


**Figure S5. Composition of oral microbiota at the genus level between AIH and HCs.** The microbial community barplot of AIH and HCs visually reflected the relative abundance of microbiome at genus level for each sample. AIH, Autoimmune hepatitis; HC, healthy controls

**Supplementary Figure 6**

**
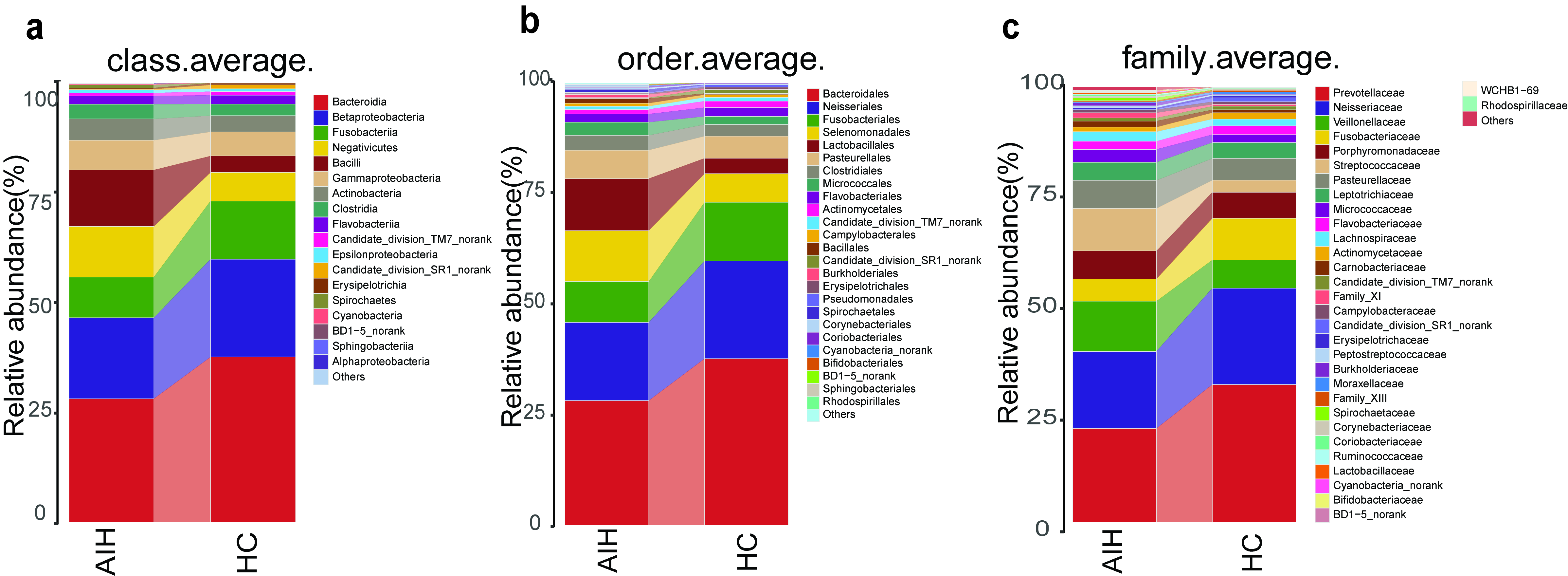
**

**Figure S6. Composition of oral microbiota on taxonomic level between AIH and HCs.** Composition of oral microbiota at (a) the class level, (b) order level and (c) family level in AIH (left) versus HCs (right). AIH, Autoimmune hepatitis; HC, healthy controls.

**Supplementary Figure 7**


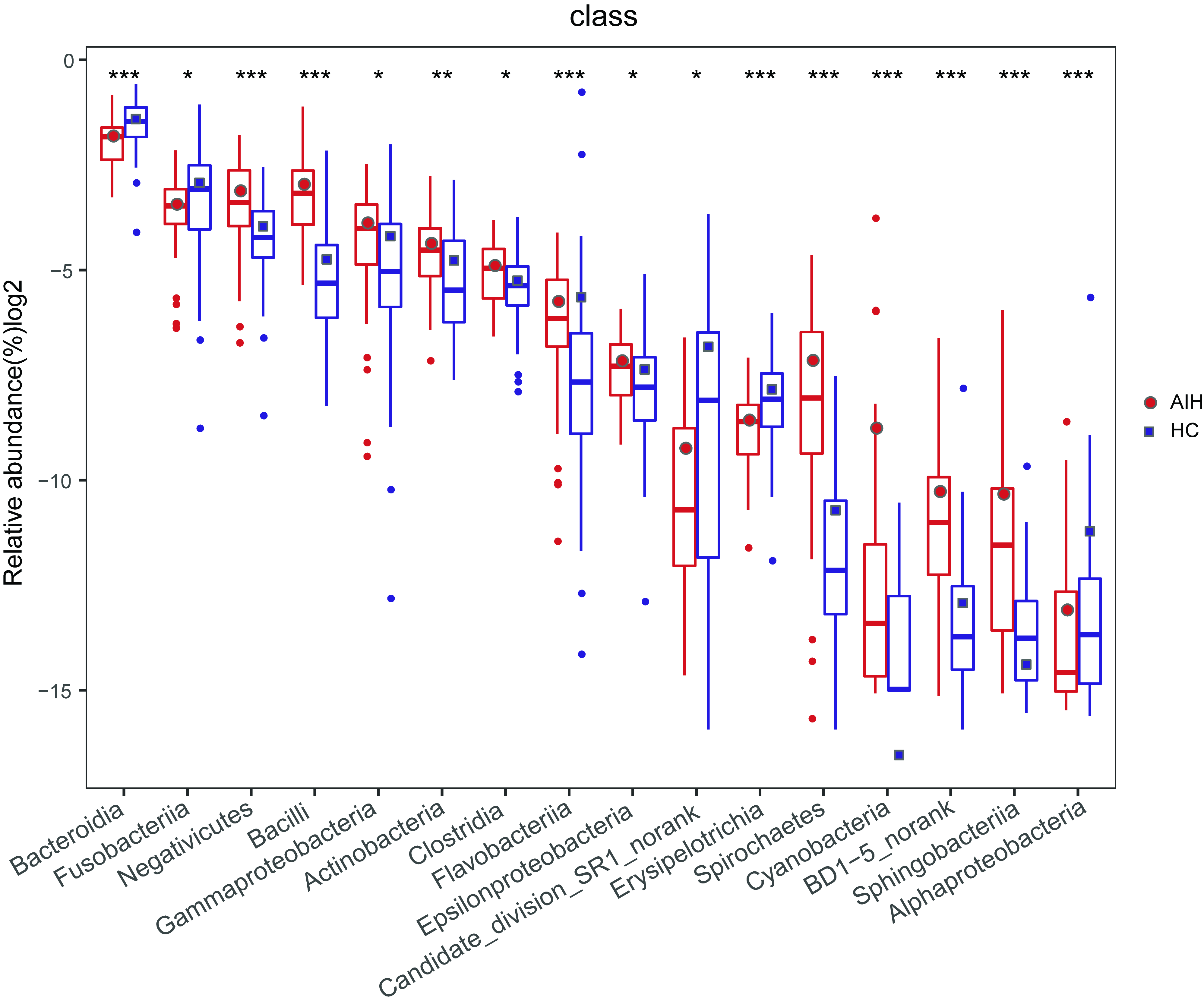


**Figure S7.** **Comparison of oral microbiome at the class level between AIH and HCs.** Comparison of oral microbiota at the class level between AIH (red) and HCs (blue). The box presented the 95% CIs; the line inside denotes the median, and the dot inside denotes the mean value. P values were calculated using the Wilcoxon rank sum test, and are shown in supplementary Data S12. Significant differences by *P < 0.05; **P <0.01 and ***P < 0.001.AIH, Autoimmune hepatitis; HC, healthy controls.

**Supplementary Figure 8**


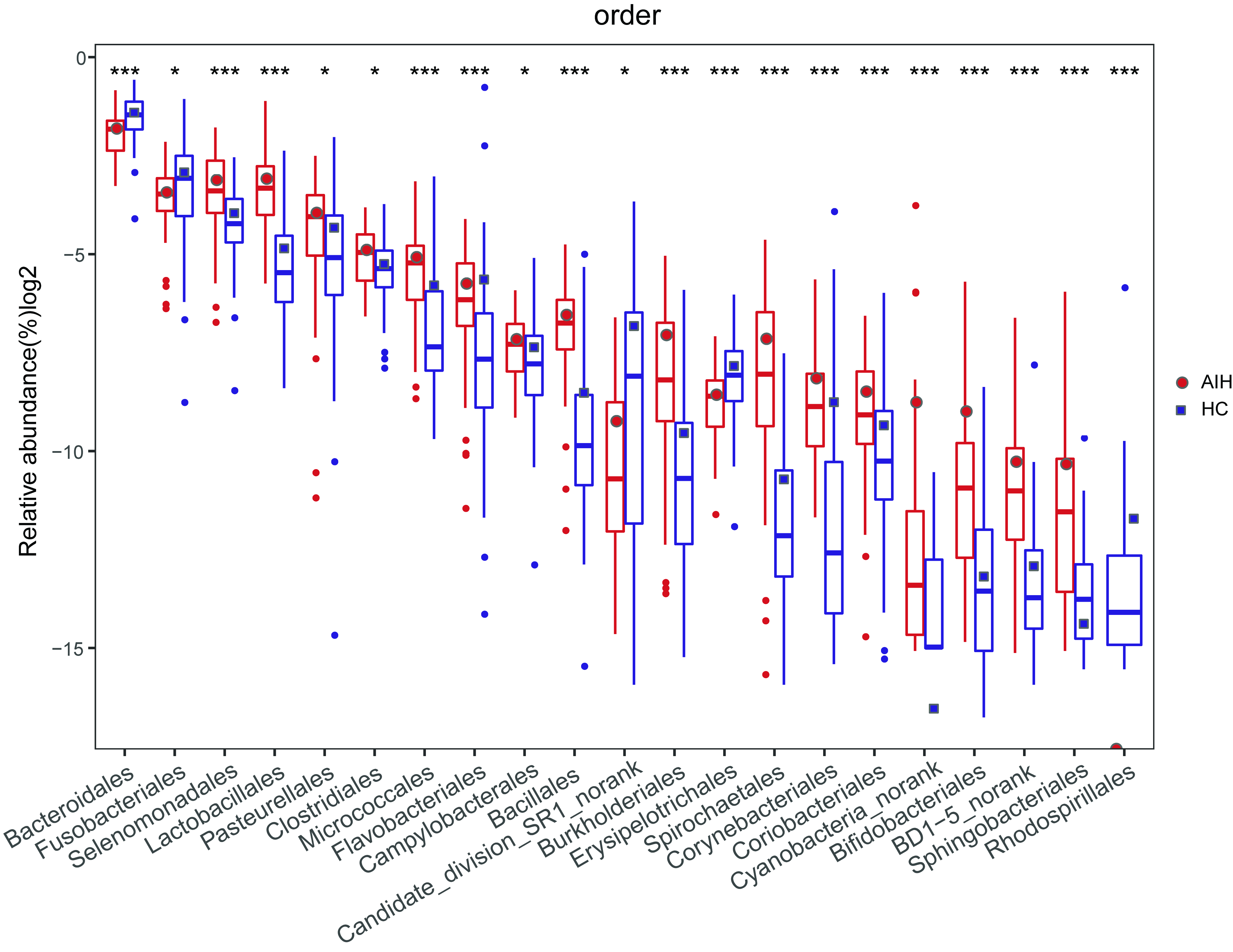


**Figure S8. Comparison of oral microbiome at the order level between AIH and HCs.** Comparison of oral microbiota at the order level between AIH (red) and HCs (blue). The box presented the 95% CIs; the line inside denotes the median, and the dot inside denotes the mean value. P values were calculated using the Wilcoxon rank sum test, and are shown in supplementary Data S13. Significant differences by *P < 0.05; **P <0.01 and ***P < 0.001. AIH, Autoimmune hepatitis; HC, healthy controls.

**Supplementary Figure 9**


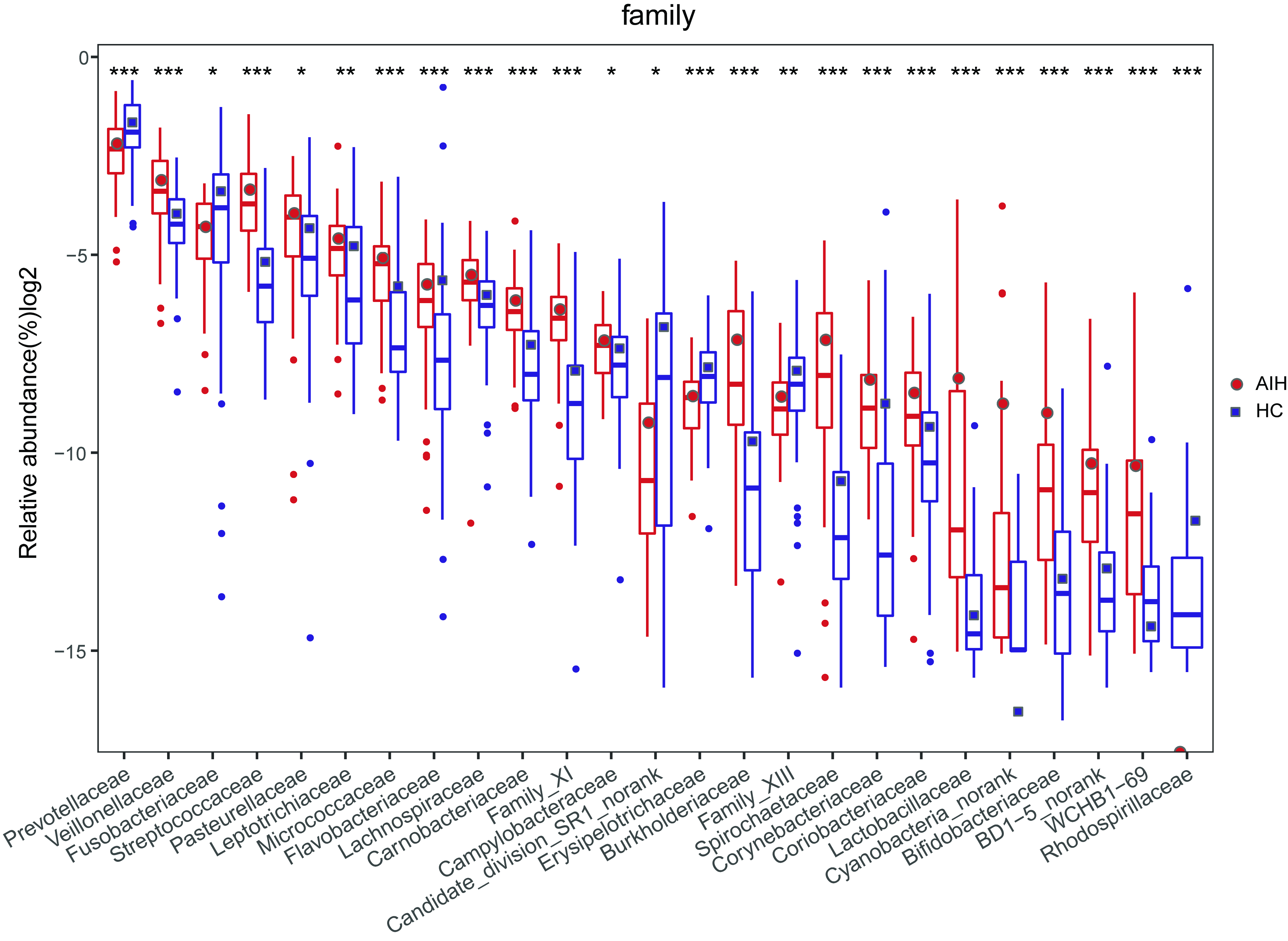


**Figure S9. Comparison of oral microbiome at the** **family level between AIH and HCs.** Comparison of oral microbiota at the family level between AIH (red) and HCs (blue). The box presented the 95% CIs; the line inside denotes the median, and the dot inside denotes the mean value. P values were calculated using the Wilcoxon rank sum test, and are shown in supplementary Data S14. Significant differences by *P < 0.05; **P <0.01 and ***P < 0.001. AIH, Autoimmune hepatitis; HC, healthy controls.
